# Kolmogorov Entropy for Convergence Rate in Incomplete Functional Time Series: Application to Percentile and Cumulative Estimation in High Dimensional Data

**DOI:** 10.3390/e25071108

**Published:** 2023-07-24

**Authors:** Ouahiba Litimein, Fatimah Alshahrani, Salim Bouzebda, Ali Laksaci, Boubaker Mechab

**Affiliations:** 1 Laboratory of Statistics and Stochastic Processes, University of Djillali Liabes, BP 89, Sidi Bel Abbes 22000, Algeria; ouahiba.litimein@univ-sba.dz (O.L.); mechaboub@yahoo.fr (B.M.); 2 Department of Mathematical Sciences, College of Science, Princess Nourah bint Abdulrahman University, P.O. Box 84428, Riyadh 11671, Saudi Arabia; fmalshahrani@pnu.edu.sa; 3 Laboratoire de Mathématiques Appliquées de Compiègne (L.M.A.C.), Université de Technologie de Compiègne, 60200 Compiègne, France; 4 Department of Mathematics, College of Science, King Khalid University, Abha 62529, Saudi Arabia; alikfa@kku.edu.sa

**Keywords:** functional data, missing data, small ball probability, local linear modeling, Kolmogorov entropy, almost complete (a.co.) convergence, 62G20, 62M10, 62N01, 62P30, 60F05

## Abstract

The convergence rate for free-distribution functional data analyses is challenging. It requires some advanced pure mathematics functional analysis tools. This paper aims to bring several contributions to the existing functional data analysis literature. First, we prove in this work that Kolmogorov entropy is a fundamental tool in characterizing the convergence rate of the local linear estimation. Precisely, we use this tool to derive the uniform convergence rate of the local linear estimation of the conditional cumulative distribution function and the local linear estimation conditional quantile function. Second, a central limit theorem for the proposed estimators is established. These results are proved under general assumptions, allowing for the incomplete functional time series case to be covered. Specifically, we model the correlation using the ergodic assumption and assume that the response variable is collected with missing at random. Finally, we conduct Monte Carlo simulations to assess the finite sample performance of the proposed estimators.

## 1. Introduction

Statistical problems associated with the study of functional random variables, that is, variables with values in an infinite-dimensional space, have garnered increasing attention in the statistical literature over the past few decades. The abundance of data measured on increasingly fine temporal/spatial grids, as is the case in meteorology, medicine, satellite imagery, and numerous other research fields, has inspired the development of this research theme. Thus, the statistical modeling of these data as random functions resulted in a number of difficult theoretical and numerical research concerns; we may refer to [1,2,3,4,5,6] for parametric and nonparametric models. For the latest contributions in FDA and its related topics, one can refer to [7,8,9,10,11,12,13,14].

Quantile regression has emerged as a significant statistical technique for data analysis since Koenker and Bassett’s [15] seminal work. But, concerns about quantile crossing and model misspecification [16,17,18] have led to the development of the nonparametric estimation of conditional quantile functions [19,20]. This estimate originates from [21], who proved the convergence using the probability of the empirical conditional law function; refer to [22,23]. This technique is an alternative to mean regression, and it possesses many desirable properties, such as being more efficient than mean regression when the data follow a heavy-tailed distribution, and it is frequently used to characterize the entire conditional distribution of the response. Conditional percentile estimation has been studied extensively by several researchers [24,25,26]. However, most of them used the kernel method approach. For instance, Ferraty et al. [4] have studied the uniform convergence of such an estimation in the analogously distributed and independent case. However, in the same case, Samanta [27] acquired the almost complete (a.co.) convergence of the conditional percentile estimation.

Studies of local linear estimation conditional quantile function (LLECQF) are still limited. For example, Messaci et al. [28] have studied the local linear estimation (LLE) of the conditional quantile function (CQF) by reversing the estimator shown in [29]. Al-Awadhi et al. [30] have proved the a.co. convergence and the asymptotic law of the CQF by considering an estimator based on the $L_{1}$ approach. Despite its importance in functional data analysis, the LLE has various merits over the kernel technique. Generally, this method can reduce the bias properties of the kernel approach [31,32]. Furthermore, the LLE has been lately introduced in the FDA by [33]. The latter concentrates on the LLE of the curve regression while the insert variable is a Hilbert class. Barrientos-Marin et al. [34] have proposed the LLE of the nonparametric regression operator and studied their asymptotic properties. In particular, this operator can be applied to functional covariable.

In this article, we are interested in studying the local linear estimation of the conditional cumulative distribution function (LLECCDF) and LLECQF under the assumption of ergodicity. We are interested in the uniform a.co. convergence of the constructed sequences using the Kolmogorov entropy function. In addition to Kolmogorov entropy, other information measures such as Kullback–Leibler divergence have been considered for a convergence rate study of estimators in multivariate time series modelling; refer to [35,36].

From a practical point of view, the ergodic framework is an essential condition in statistical physics, number theory, Markov chains, and other fields. The concept of ergodicity is fundamental in the research of stochastic processes. Note also that one of the arguments invoked by [37] motivating the introduction of the concept of ergodicity is that, for certain classes of processes, it can be much easier to prove ergodic properties rather than the mixing condition. Hence, the ergodicity hypothesis seems to be the most naturally adapted and provides a better framework to study data series such as those generated by noisy chaos.

In their discussion, [38] provided an example of processes that are ergodic but not mixing, which may be summarized as follows: let $\left\{\left(T_{i},\lambda_{i}\right):i\in Z\right\}$ be a strictly stationary process such that $T_{i}|T_{i-1}$ is a Poisson process with a parameter $\lambda_{i}$, where $T_{i}$ is the σ-field generated by $\left(T_{i},\lambda_{i},T_{i-1},\lambda_{i-1},\ldots \right)$. Assume that $\lambda_{i}=f\left(\lambda_{i-1},T_{i-1}\right)$, where f:[0,∞)×N→(0,∞) is a given function. This process does not mix in general (see Remark 3 of [39]). It is known that any sequence ${\left(\varepsilon_{i}\right)}_{i\in Z}$ of i.i.d. random variables is ergodic. Hence, it is immediately clear that ${\left(Y_{i}\right)}_{i\in Z}$ with $Y_{i}=\vartheta \left(\left(\ldots ,\varepsilon_{i-1},\varepsilon_{i}\right),\left(\varepsilon_{i+1},\varepsilon_{i+2},\ldots \right)\right),$ for some Borel-measurable function ϑ(·), is also ergodic; see Proposition 2.10 on page 54 in [40]. Under the condition of ergodicity, [41] have studied the conditional quantile for ergodic data by considering an iterative model. However, under the same conditions, the authors in [42] have considered the nonparametric estimation of quantile for censored data.

All these studies were concerned with the complete-data situation. This work investigates the question of when data are missing at random (MAR); for instance, see [43]. In contemporary statistics, missing data are pervasive, posing a significant obstacle for various applications. The concept of missing data in statistics occurs when a data value of the variable is not stored in the observation. For example, missingness may occur when our data are compiled from sources that have measured various variables; for instance, in the healthcare industry, the data routinely collected on patients may vary between clinics and hospitals. Among numerous other reasons are sensor failure, data censorship, privacy concerns, pharmaceutical tracing tests, and reliability tests. It can occur in any experimental setting where contamination of the treatment or subject mortality is possible. This topic has been extensively examined in numerous statistical problems; see [43,44] for a thorough overview. In the present work, we only observe *Y* in cases where some indicator *B* equals one and the indicator *B* is conditionally independent of *Y* given *X*. This assumption is useful when information in the form of covariate data is available to explain the missingness; refer to [45,46,47]. The first studies on MAR are presented by [48]. They established an approximation of the regression operator and studied their asymptotic consistency when the curve regressor is observed and the interest response is missing at random. However, in the ergodic data case, Ling et al. [49] have studied the asymptotic distribution of the estimator, which is proposed in [48]; also, refer to [50,51].

The main objective of this paper is to evaluate the convergence velocity of some functional estimators through the Kolmogorov entropy function. More precisely, we focus on the local linear smoothing of the distribution function and its inverse, the quantile function. The constructed estimators’ asymptotic properties are evaluated when the data are correlated as functional ergodic time series data and the response variable is observed under the MAR structure. The efficiency of these estimators is uniformly specified using the entropy metric, allowing us to assess the impact of the functional path of the data. In particular, the Kolmogorov entropy gives a trade-off between the data’s sparsity and the approximation’s efficiency. Moreover, the Kolmogorov entropy explores the functional space’s topological structure and its spectral property. Thus, stating the uniform consistency concerning the Kolmogorov entropy is more beneficial than the classical pointwise case. Although the uniform convergence of LLECCDF and LLECQF in the functional ergodic time series (FETS) structure is purely mathematically challenging, the obtained results are also pivotal for many applied issues, such as the smoothing parameter choice, bootstrapping, and the single index modeling. The second challenging issue of this contribution concerns the MAR feature of the response variable. Our results can be used to determine an estimator of the unconditional distribution of the scalar response, even if it is not completely observed. All these challenging issues will be discussed using specific examples in Section 6. In addition to the uniform consistency, we prove the asymptotic normality of the LLECCDF, which is important to provide a confidence interval comparable with the predictive interval deduced from LLECQF. Once again, this prediction using the predictive subset is also primordial in the context of incomplete functional time series data. Finally, we point out that, to our best knowledge, this problem of uniform consistency of local linear approximation under MAR and FETS structures was open up to the present, giving the main motivation to our paper.

The rest of the paper is organized as follows. In Section 2, we state the formal setup and define the estimators. More precisely, Section 2.1 is devoted to the LLECCDF estimator, while Section 2.2 introduces the LLECQF estimator. The convergence rate of the two approximation sequences is established in Section 3. In Section 4, we derive the limiting distribution of the proposed estimators. In Section 5, we conduct Monte Carlo simulations to assess the finite sample performance of the proposed estimators. Section 6 is devoted to highlighting the principal features of our contribution. In Section 7, we give some concluding remarks. All the proofs are gathered in the last section.

## 2. Model and Estimators

### 2.1. LLECCDF: Numerical Approximation of CCDF-Model

Let $\left\{\left(X_{i},Y_{i}\right):1\leq i\leq n\right\}$ be a sequence of stationary ergodic functional random variables identically distributed as (X,Y), where *X* takes values in a some semi-metric abstract space F with a semi-metric d(·,·) and *Y* takes values in R. For reader convenience, we introduce some details defining the ergodic property of processes and its link with the mixing one. Let $\left\{X_{n},n\in Z\right\}$ be a stationary sequence. Consider the backward field $A_{n}=\sigma \left(X_{k}:k\leq n\right)$ and the forward field $B_{m}=\sigma \left(X_{k}:k\geq m\right)$. The sequence is strongly mixing if

$$\underset{A\in A_{0},B\in B_{n}}{\sup}\left|P\left(A\cap B\right)-P\left(A\right)P\left(B\right)\right|\to 0,\;\;\mathrm{as}\;\;n\to \infty .$$

The sequence is ergodic if, for any two measurable sets A,B,

$$\lim_{n\to \infty }\frac{1}{n}\Sigma_{k=0}^{n-1}\left|P\left(A\cap \tau^{-k}B\right)-P\left(A\right)P\left(B\right)\right|=0,$$

where τ is the time evolution or shift transformation taking $X_{k}$ into $X_{k+1}$. We shall also use the same symbol τ to denote the induced set transformation, which takes, for example, sets $B\in B_{m}$ into sets $\tau B\in B_{m+1}$; for instance, see [52]. The naming of strong mixing in the above definition is more stringent than what is ordinarily referred to (when using the vocabulary of measure preserving dynamical systems) as strong mixing, namely to that $\lim_{n\to \infty }P\left(A\cap \tau^{-n}B\right)=P\left(A\right)P\left(B\right)$ for any two measurable sets A,B; see, for instance [52,53] and more recent references [54,55,56,57,58,59,60]. Hence, strong mixing implies ergodicity, whereas the inverse is not always true (see, e.g., Remark 2.6 in page 50 in connection with Proposition 2.8 in page 51 in [40]). For every x∈F, the function of conditional law CFD(y|x) of *Y* when X=x is defined by

$$CFD\left(y|x\right)=P\left(Y\leq y|X=x\right).$$

The LLECCDF is obtained by assuming for every *z* in the vicinity of *x*

(1) $$CDF\left(y|z\right)=\beta_{1}+\beta_{2}\alpha \left(z,x\right)+o\left(\alpha \left(z,x\right)\right)\;with\;\alpha \left(z,z\right)=0,$$

where α(·,·) is a bilinear locating function such that

 **(i)** 
For all $x^{\prime }\in C_{F}$, $C^{\prime }|\delta \left(x,x^{\prime }\right)|\leq |\alpha \left(x^{\prime },x\right)|\leq C|\delta \left(x,x^{\prime }\right)|;$
 **(ii)**  For all $z_{1},z_{2}\in C_{F}$, $|\alpha \left(z_{1},x\right)-\alpha \left(z_{2},x\right)|\leq C^{{}^{\prime }}\left|\delta \left(z_{1},z_{2}\right)\right|$,

where δ(·,·) is a bilinear function such that |δ(·,·)|=d(·,·). In the rest of the paper, we suppose that the CDF is of the $C^{1}$-class with respect to *y* and its derivative is the conditional density function denoted by cdf(·). However, in the case of a missing response, one has an incomplete sample of size *n* from (X,Y,B), which is usually denoted by $\left\{\left(X_{i},Y_{i},B_{i}\right):1\leq i\leq n\right\}$, where $B_{i}=1$ if $Y_{i}$ is observed, and $B_{i}=0$ otherwise. The Bernoulli random variable *B* is supposed to be such that

P(x)=P(B=1|X=x)=P(B=1|X=x,Y=y)

is a continuous function. Under this smoothing consideration, we define the LLECCDF of CDFF(·∣·) by finding the minimizers $\left(\hat{\beta_{1}},\hat{\beta_{2}}\right)$ of

(2) $$\begin{array}{c} \min_{\left(\beta_{1},\beta_{2}\right)\in R^{2}}\Sigma_{i=1}^{n}{\left(J\left(\frac{y-Y_{i}}{\lambda_{J}}\right)-\beta_{1}-\beta_{2}\alpha \left(X_{i},x\right)\right)}^{2}Ker\left(\frac{\delta (x,X_{i})}{\lambda_{K}}\right)B_{i}, \end{array}$$

where $\lambda_{K}:=\lambda_{K,n}$ and $\lambda_{J}:=\lambda_{J,n}$ are bandwidth parameters and J(·) and Ker(·) are, respectively, distribution and kernel functions. The explicit solution to this minimization is

$$\hat{\beta_{1}}=\hat{CDF}\left(y|x\right)=\frac{\Sigma_{j=1}^{n}\Gamma_{j}\left(x\right)Ker_{j}\left(x\right)B_{j}J_{j}\left(y\right)}{\Sigma_{j=1}^{n}\Gamma_{j}\left(x\right)B_{j}Ker_{j}\left(x\right)},$$

with

$$\begin{array}{ccc} \Gamma_{j}\left(x\right) & = & \Sigma_{i=1}^{n}\alpha_{i}^{2}\left(x\right)Ker_{i}\left(x\right)B_{i}-\left(\Sigma_{i=1}^{n}\alpha_{i}\left(x\right)Ker_{i}\left(x\right)B_{i}\right)\alpha_{j},\\ \;Ker_{j}\left(x\right) & = & Ker\left(\frac{\delta (x,X_{j})}{\lambda_{K}}\right) \end{array}$$

and

$$J_{j}\left(y\right)=J\left(\frac{y-Y_{j}}{\lambda_{J}}\right),\;\alpha_{j}\left(x\right)=\alpha \left(X_{j},x\right).$$

Furthermore, we can write

$$\hat{CDF}\left(y|x\right)=\frac{{\hat{CDF}}_{N}^{x}\left(y\right)}{{\hat{CDF}}_{D}\left(x\right)},$$

where

$${\hat{CDF}}_{N}^{x}\left(y\right):=\frac{1}{nI\;E(\Gamma_{1}\left(x\right)Ker_{1}\left(x\right))}\Sigma_{j=1}^{n}\Gamma_{j}\left(x\right)Ker_{j}\left(x\right)J_{j}\left(y\right)B_{j},$$

and

$${\hat{CDF}}_{D}\left(x\right):=\frac{1}{nI\;E(\Gamma_{1}\left(x\right)Ker_{1}\left(x\right))}\Sigma_{j=1}^{n}\Gamma_{j}\left(x\right)Ker_{j}\left(x\right)B_{j}.$$

The first main contribution of this work is a precise convergence rate of the approximation $\hat{CDF}\left(\cdot |\cdot \right)$ uniformly over a non-necessary compact subset $C_{F}$ of F. We use the notation

$$Ba\left(x,h\right)=\left\{x^{\prime }\in F:d\left(x^{\prime },x\right)\leq h\right\}.$$

**Definition** **1.** *Let $C_{F}$ be a subset of a semi-metric space F, and let ε>0 be given. A finite set of points $x_{1},x_{2},\ldots ,x_{N}$ in F is called an ε-net for $C_{F}$ if*

$$C_{F}\subset \overset{N}{\underset{k=1}{⋃}}Ba\left(x_{k},\varepsilon \right).$$

*The quantity $\psi_{C_{F}}\left(\varepsilon \right)=\log\left(N_{\varepsilon }\left(C_{F}\right)\right)$, where $N_{\varepsilon }\left(C_{F}\right)=:d_{n}$ is the minimal number of open balls in F of radius ε which is necessary to cover $C_{F}$, is called Kolmogorov’s ε-entropy of the set $C_{F}$.*

This concept was introduced by Kolmogorov in the mid-1950s (refer to [61]). It serves as a measure of the complexity of a set, indicating that high entropy implies that a significant amount of information is required to accurately describe an element within a given tolerance, ε. Consequently, the selection of the topological structure (specifically, the choice of semi-metric) plays a crucial role when examining asymptotic results that are uniform over a subset, $C_{F}$ of F. In particular, we subsequently observe that a well-chosen semi-metric can enhance the concentration of the probability measure for the functional variable, *X*, while minimizing the ε-entropy of the subset, $C_{F}$. Ferraty and Vieu [6] emphasized the phenomenon of concentration of the probability measure for the functional variable by calculating small ball probabilities in different standard scenarios; refer to [62]. For readers interested in these concepts (entropy and small ball probabilities) and/or the utilization of Kolmogorov’s ε-entropy in dimensionality reduction problems, we recommend referring to [63] or/and [64], respectively.

Let $\left(u_{n}\right)$ for n∈N, be a sequence of real r.v.s. We say that $\left(u_{n}\right)$ converges almost-completely (a.co.) toward zero if, and only if, for all ϵ>0,

$$\Sigma_{n=1}^{\infty }P\left(\left|u_{n}\right|>\epsilon \right)<\infty .$$

Moreover, we say that the rate of the almost-complete convergence of $\left(u_{n}\right)$ toward zero is of order $v_{n}$ (with $v_{n}\to 0$), and we write $u_{n}=O_{a.co.}\left(v_{n}\right)$ if, and only if, there exists ϵ>0 such that

$$\Sigma_{n=1}^{\infty }P\left(\left|u_{n}\right|>\epsilon v_{n}\right)<\infty .$$

This kind of convergence implies both the almost-sure convergence and the convergence in probability.

### 2.2. LLECQF: Numerical Approximation of CQF-Model

The second approximation concerns the CQF-Model of order *p*, for p∈(0,1), denoted as $CQF_{p}\left(x\right)$:

$$CDF(CQF_{p}\left(x\right)|x)=p.$$

The natural estimator is obtained by inverting the LLECCDF. However, unlike the local constant estimator, the LLECCDF is not monotone and invertible. To overcome this issue, we use the robust definition of the $CQF_{p}\left(x\right)$:

(3) $$CQF_{p}\left(x\right)=\arg\min_{t}I\;E\left[L_{p}\left(Y-t\right)\;|X=x\right],$$

where the scoring function $L_{p}\left(y\right)=\left|y\right|\left(p-11_{\left[y<0\right]}\right)$, with $11_{S}$ as the indicator of *S*. Once again, the LLECQF is obtained via a smoothing approximation of the model locally in the neighborhood of the location *x*. Therefore, we suppose that $CQF_{p}\left(x\right)$ is such that

(4) $$CQF_{p}\left(z\right)=\beta_{1}+\beta_{2}\alpha \left(z,x\right)+o\left(\alpha \left(z,x\right)\right).$$

Therefore, $\beta_{1}$ and $\beta_{2}$ are estimated as

(5) $$\arg\min_{\left(\beta_{1},\beta_{2}\right)\in I\;R^{2}}\Sigma_{j=1}^{n}L_{p}\left(Y_{j}-\beta_{1}-\beta_{2}\alpha \left(X_{j},x\right)\right)Ker_{j}\left(x\right)B_{j}.$$

So, the LLECQF of $CQF_{p}\left(x\right)$ is denoted by

$$\hat{CQF_{p}}\left(x\right)=\tilde{\beta_{1}},$$

where

$$\left(\tilde{\beta_{1}},\tilde{\beta_{2}}\right)=\arg\min_{\left(\beta_{1},\beta_{2}\right)\in I\;R^{2}}\Sigma_{j=1}^{n}L_{p}\left(Y_{j}-\beta_{1}-\beta_{2}\alpha \left(X_{j},x\right)\right)Ker_{j}\left(x\right)B_{j}.$$

Once again, our main focus is to establish the convergence rate using the Kolmogorov entropy and the uniform-Lipschitzian condition: For all $\left(y_{1},y_{2}\right)\in N_{y}^{2}$ and $x_{1},x_{2}\in C_{F}$, we have

(6) $$\left|CDF^{\left(j\right)}\left(y_{1}|x_{1}\right)-CDF^{\left(j\right)}\left(y_{1}|x_{2}\right)\right|\leq C_{2}\left(|\delta \left(x_{2},x_{1}\right){|}^{k_{2}}+{\left|y_{2}-y_{1}\right|}^{k_{1}}\right),$$

for j=0,1 and $C_{2}$, $\;k_{1},k_{2}>0.$

## 3. Uniform Convergence Rate

Let $F_{i}$ and $G_{i}$(i=1,…,n) be the σ-algebras generated by $\left(\left(Y_{1},X_{1}\right),\ldots ,\left(Y_{i},X_{i}\right)\right)$ and $\left(\left(Y_{1},X_{1}\right),\ldots ,\left(Y_{i},X_{i}\right),X_{i+1}\right)$, respectively. Let $N_{y}$ denote a fixed neighborhood of a point *y* in IR. Define

$$\psi_{x}\left(\lambda_{1},\lambda_{2}\right)=I\;P\left(\lambda_{2}\leq \delta \left(X,x\right)\leq \lambda_{1}\right).$$

The function $\psi_{x}\left(\cdot ,\cdot \right)$ plays a similar role to small ball probability as in [34]. Finally, let *C* and $C^{{}^{\prime }}$ be some generic constants that are strictly positive. To obtain the convergence rate in this functional ergodic MAR scheme, we consider the following assumptions:

(H1)  The first-order derivative of ψ(·) exists and is bounded in $C_{F}$. The function ψ(·) is such that
  $$0<C^{\prime }\psi \left(\varsigma \right)\leq \underset{x\in C_{F}}{\inf}I\;P\left(X\in Ba\left(x,\varsigma \right)\right)\leq \underset{x\in C_{F}}{\sup}I\;P\left(X\in Ba\left(x,\varsigma \right)\right)\leq C\psi \left(\varsigma \right).$$
(H2)  There exists a non-random function $\psi_{i}\left(\cdot \right)$ satisfying
   **(i)**     $0<C\psi_{i}\left(\varsigma \right)<I\;P\left(X_{i}\in Ba\left(x,\varsigma \right)|F_{i-1}\right)\leq C^{{}^{\prime }}\psi_{i}\left(\varsigma \right)$,
   **(ii)**   $\frac{1}{n\psi (\varsigma )}\Sigma_{i=1}^{n}\psi_{i}\left(\varsigma \right)⟶1,a.\mathrm{co}.,$
   **(iii)**  There exists a non-random function Ψ(·) satisfying
    $$\forall t\in \left[-1,1\right],\;\lim_{\lambda_{K}\to 0}\frac{\psi (t\lambda_{K})}{\psi (\lambda_{K})}=\Psi \left(t\right).$$
(H3)  The distribution function J(·) and the kernel Ker(·) fulfill the following:
   **(i)**  Ker(·) is a Lipschitz on its support [−1,1] satisfying
    $$D=\left(\begin{array}{cc} Ker\left(1\right)-\int_{-1}^{1}Ker^{\prime }\left(t\right)\Psi \left(t\right)dt & Ker\left(1\right)-\int_{-1}^{1}{\left(tKer\left(t\right)\right)}^{\prime }\Psi \left(t\right)dt\\ Ker\left(1\right)-\int_{-1}^{1}{\left(tKer\left(t\right)\right)}^{\prime }\Psi \left(t\right)dt & Ker\left(1\right)-\int_{-1}^{1}{\left(t^{2}Ker\left(t\right)\right)}^{\prime }\Psi \left(t\right)dt \end{array}\right)\;,$$
    is a positive definite matrix.
   **(ii)**  The function J(·) has a derivative satisfying
    $${\int |t|}^{k_{1}}J^{\left(1\right)}\left(t\right)dt<\infty .$$
   **(iii)**  We almost surely have $I\;E\left[J_{j}\left(y\right)|G_{j-1}\right]=I\;E\left[J_{j}\left(y\right)|X_{j}\right].$
(H4)  The real sequence $d_{n}$ associated with $r_{n}=O\left(\frac{\ln n}{n}\right)$ satisfies
  $$\frac{{\left(\ln n\right)}^{2}}{n\psi (\lambda_{K})}<\ln d_{n}<\frac{n\psi (\lambda_{K})}{\ln n}\;\;\mathrm{and}\;\;\Sigma_{n=1}^{\infty }d_{n}^{1-\varrho }<\infty \;\mathrm{for}\;\;\mathrm{some}\;\;\varrho >1.$$
(H5)  The bandwidth $\lambda_{K}$ is linked to α(·) and ψ(·) by
   **(i)**  $$\forall n>n_{0}\;-\frac{1}{\psi (\lambda_{K})}\int_{-1}^{1}\psi \left(z\lambda_{K},\lambda_{K}\right)\frac{d}{dz}\left(z^{2}(Ker\left(z\right)\right)dz>C;$$
   **(ii)**  $\lim_{n\to \infty }\lambda_{K}=0,$ and $\lim_{n\to \infty }\lambda_{H}=0,$ and $\lim_{n\to \infty }\frac{\ln n}{n\psi (\lambda_{K})}=0;$
   **(iii)**  $$\lambda_{K}\int_{Ba(x,\lambda_{K})}\alpha \left(u,x\right)dP\left(u\right)=O\left(\int_{Ba(x,\lambda_{K})}\alpha^{2}\left(u,x\right)dP\left(u\right)\right),$$
    where P is the law of *X*.

We first recall that our assumptions are not restrictive and may be considered as standard in the functional local linear analysis context. They are similar to those used in the local linear estimation of the quantile regression in [30]. In particular, Assumption (H1) concerns the usual concentration property of the functional variable. It is well documented that this property allows us to explore the functionality nature of the data. Assumption (H3)(iii) is a Markov-type condition and characterizes the conditional moments. It is satisfied when considering, for instance, the model

$$11_{\left(Y\leq y\right)}=m_{\psi }\left(X_{i}\right)+\epsilon_{i},$$

where $\left(\epsilon_{i}\right)$ is a square-integrable process independent of $\left(X_{i}\right)$. Finally, Assumptions (H3)(i)-(ii), (H4), and (H5) are technical conditions, similar to those used by [30].

The following theorem gives the uniform, almost complete convergence of $\hat{CDF}\left(y|x\right)$ with the rate.

**Theorem** **1.** *Under Conditions (H1)–(H5), we have*

(7) $$\underset{x\in C_{F}}{\sup}\left|\hat{CDF}\left(y|x\right)-CDF\left(y|x\right)\right|=O\left(\lambda_{K}^{k_{1}}+\lambda_{J}^{k_{2}}\right)+O_{a.co.}\left({\left(\frac{\ln d_{n}}{n\psi (\lambda_{K})}\right)}^{\frac{1}{2}}\right).$$

The proof of Theorem 1 is postponed until the last section. The following theorem gives the uniform, almost complete convergence of $\hat{CQF_{\alpha }}\left(x\right)$ with the rate.

**Theorem** **2.** *Under Conditions (H1)–(H5) and if $cdf(CQF_{\alpha }\left(x\right)|x)>0$, we have*

(8) $$\underset{x\in C_{F}}{\sup}\left|\hat{CQF_{\alpha }}\left(x\right)-CQF_{\alpha }\left(x\right)\right|=O\left(\lambda_{K}^{\min(k_{2},k_{2})}\right)+O_{a.co.}\left({\left(\frac{\ln d_{n}}{n\psi (\lambda_{K})}\right)}^{\frac{1}{2}}\right).$$

The proof of Theorem 2 is postponed until the last section.

## 4. Asymptotic Normality

The second asymptotic result concerns the asymptotic law of the sequence $\hat{CDF}\left(y|x\right)$. To do that, we enhance Assumption (H5) by assuming that the kernel Ker(·) satisfies (H4) and has a first derivative $Ker^{{}^{\prime }}\left(\cdot \right)$ such that

$$Ker^{2}\left(1\right)-\int_{-1}^{1}{\left(Ker^{2}\left(u\right)\right)}^{{}^{\prime }}\Psi \left(u\right)du>0.$$

Let us now state the following theorem, which gives the central limit theorem of the estimator $\hat{CDF}\left(y|x\right)$. Below, we write $Z\overset{D}{=}N\left(\mu ,\sigma^{2}\right)$ whenever the random variable *Z* follows a normal law with expectation μ and variance $\sigma^{2}$; $\overset{D}{\to }$ denotes the convergence in distribution.

**Theorem** **3.** *Under the assumptions of Theorem 1 and if the smoothing parameters $\lambda_{K}$ satisfy $\lim_{n\to \infty }n\lambda_{K}^{2k_{1}}\psi \left(\lambda_{K}\right)=0$, then*

$$\sqrt{n\psi (\lambda_{K})}\left(\hat{CDF}\left(y|x\right)-CDF\left(y|x\right)\right)\overset{D}{\to }N\left(0,V_{JK}\left(y|x\right)\right),$$

*where*

$$M_{i}=Ker^{i}\left(1\right)-\int_{-1}^{1}{\left(Ker^{i}\left(u\right)\right)}^{{}^{\prime }}\Psi \left(u\right)du,$$

*for i=1,2 and*

$$V_{JK}\left(y|x\right)=\frac{M_{2}}{M_{1}^{2}}P\left(x\right)CDF\left(y|x\right)\left(1-CDF\left(y|x\right)\right).$$

The proof of Theorem 3 is postponed until the last section.

Clearly, this asymptotic result has many applications in practice. In particular, it can be used to build a confidence interval for the true value of CDF(y∣x). The latter is obtained by estimating the asymptotic variance $V_{JK}\left(y|x\right)$ using a plug-in approach. Indeed, $M_{1}$ and $M_{2}$ are estimated via

$$\hat{M_{1}}=\frac{1}{n\psi (\lambda_{K})}\Sigma_{i=1}^{n}Ker_{i}\left(x\right),\;\hat{M_{2}}=\frac{1}{n\psi (\lambda_{K})}\Sigma_{i=1}^{n}Ker_{i}^{2}\left(x\right),$$

and

$$\hat{P(x)}=\frac{\Sigma_{i=1}^{n}B_{i}Ker_{i}\left(x\right)}{\Sigma_{i=1}^{n}Ker_{i}\left(x\right)}.$$

Therefore, the natural estimator of the asymptotic variance $V_{JK}\left(y|x\right)$ is

$$\hat{V_{JK}}\left(y|x\right)=\frac{{\hat{M}}_{2}}{{\hat{M}}_{1}^{2}}\hat{P(x)}\hat{CDF}\left(y|x\right)\left(1-\hat{CDF}\left(y|x\right)\right).$$

Under this consideration, the $\left(1-\zeta \right)$ confidence interval for CDF(y∣x) is

$$\hat{CDF}\left(y|x\right)\pm t_{1-\zeta /2}\times {\left(\frac{\hat{V_{JK}}\left(y|x\right)}{n\psi (\lambda_{K})}\right)}^{1/2},$$

where $t_{1-\zeta /2}$ denotes the 1−ζ/2 quantile of the standard normal distribution. We note that the function ψ(·) does not appear in the calculation of the confidence interval since it will be simplified.

## 5. Numerical Results

In this computational study, we illustrate the three fundamental axes of our topic that are the functional structure, the asymptotic normality, and the local linear smoothing. In the first illustration, we examine the functional structure’s impact on the constructed estimators’ convergence rate. To cover the general features of our study, the ergodicity, and the MAR features, we generate an artificial functional time series using the Hilberthian autoregressive processes. Of course, this kind of process’s linearity allows us to incorporate our theoretical assumption concerning the ergodicity structure. To do this, we employ the routine-code *fts.rar* from the R package *freedom.fda*. The latter has a nice feature that is based on the dynamic functional principal component analysis (DFPCA); for instance, see [65]. In this empirical study, we use DFPCA to generate the ergodic function using specific basis functions. Specifically, we have used the Fourier basis functions (FBF) to obtain the functional $\left(X_{i}\right)$. Formally,

$$X_{i}=\Upsilon \left(X_{i-1}\right)+\varepsilon_{i},$$

where Υ is an operator with kernel ψ(·,·) and $\varepsilon_{i}$ is the white noise. The kernel operator is expressed by

$$\Upsilon \left(X\left(\cdot \right)\right)=\int_{0}^{1}\psi \left(t,s\right)X\left(s\right)ds.$$

**Figure 1:**
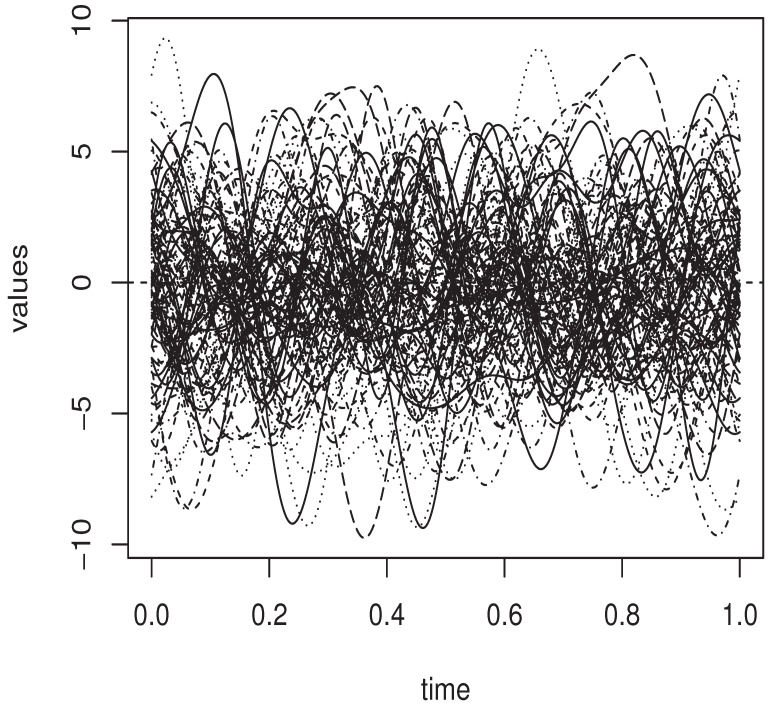
A sample of 100 curves.

In the cited routine code Υ is constructed from FBF $\left\{u_{j}:j=1,\ldots ,d\right\}$ by taking ${\left(\psi_{ij}\right)}_{ij}=\left(\langle \Psi \left(u_{i}\right),u_{j}\rangle \right)$ as the corresponding matrix. The function *op.norms* controls the degree of dependency in these functional observations for practical purposes. The functional observed regressors are plotted in Figure 1.

Specifically, such a sample was obtained by taking arbitrary, values allowing it to cover various degrees of ergodicity. Secondly, the variable of interest is generated using the regression equation

$$Y=\exp\left\{-\int_{0}^{1}\frac{1}{1+X^{2}\left(t\right)}dt\right\}+\epsilon ,$$

where ϵ is drown from N(0,0.5). With this consideration, the conditional law of *Y* given *X* is a normal distribution with a mean equal

$$\exp\left\{-\int_{0}^{1}\frac{1}{1+X^{2}\left(t\right)}dt\right\}.$$

The missing feature is controlled using the conditional probability of observation:

$$P\left(X=x\right)=\mathrm{expit}\left(\frac{2\gamma }{\pi }\int_{0}^{2\pi }x^{2}\left(t\right)dt\right),$$

where

$$\mathrm{expit}\left(u\right)=e^{u}/\left(1+e^{u}\right).$$

Under this consideration, the scalar γ controls the missing rate. Once again, we simulate with several values of γ to evaluate this characteristic’s influences in the estimators. The sequences LLECCDF and LLECQF are computed using the (−1,1)-quadratic kernel and the locally cross-validation on the number of neighborhoods using the mean square error (MSE)

$$\mathrm{MSE}=\frac{1}{n}\Sigma_{i=1}^{n}{\left(Y_{i}-{\hat{CQF_{0.5}}}^{-i}\left(X_{i}\right)\right)}^{2},$$

with ${\hat{CQF_{0.5}}}^{-i}$ referring to the estimation leave-one-out-curve of the conditional median. Clearly, the Kolmogorov entropy is an important factor of the topological structure. The latter is controlled through the locating functions α and δ. For the sake of shortness, we simulate α=δ equal to the $L_{2}$-distance using three between the *q*th derivatives of the curves based on the basis function, as well as the PCA-semi metric associated with the eigenfunctions of the empirical version covariance operator. Exactly, we compute this metric using the *m* first one. The MSE was evaluated over various values of *q* and *m*.

In Table 1 and Table 2, we summarize the MSE of both estimators for various values of mentioned parameters, the level of dependency (*op.norms*), the missing rate γ, and the metric parameter *q* or *m*.

It is clear that the behavior of the estimator is strongly impacted by different parameters of the estimators, including the dependency-level, the missing rate, and the topological structure. Without surprise, the topological structure has an important role. In a sense, the variability of the MSE as a function of the metric type is more important than the other parameters. All in all, the effectiveness of the estimator is also affected by the quantities (*op.norms*), γ, and *q* or *m*. In conclusion, we can say that the computational study confirms the theoretical statement that the convergence rate is strongly affected by the topological structure of the functional regressor.

The second illustration concerns the quality of the asymptotic normality result in Theorem 3. Specifically we aim to examine the behavior of the asymptotic distribution with respect to the degree of correlation, as well as the missing rate. For this purpose, we repeat the previous sampling processes independently *m* times, and for each time, we calculate the quantity:

(9) $$\sqrt{\frac{n\psi \left(\lambda_{K}\right){\hat{M}}_{1}^{2}}{{\hat{M}}_{2}\hat{P(x)}CDF\left(y|x\right)\left(1-CDF\left(y|x\right)\right)}}\left(\hat{CDF}\left(y|x\right)-CDF\left(y|x\right)\right).$$

Recall that the true conditional law of *Y* given *X* is known justifying the use of the true conditional cumulative distribution function of CDF(y∣x). Observe also that the estimation of the function $\psi (\lambda_{K})$ is not necessary. It will be simplified using the definition of ${\hat{M}}_{1}$ and ${\hat{M}}_{2}$ (see their definitions in Section 4).

**Figure 2:**
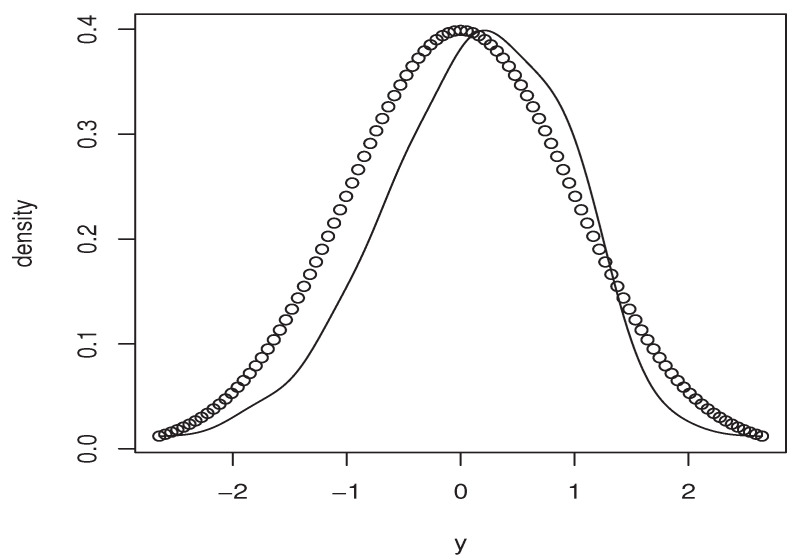
Case (op.norms,γ)=(0.98,5).

**Figure 3:**
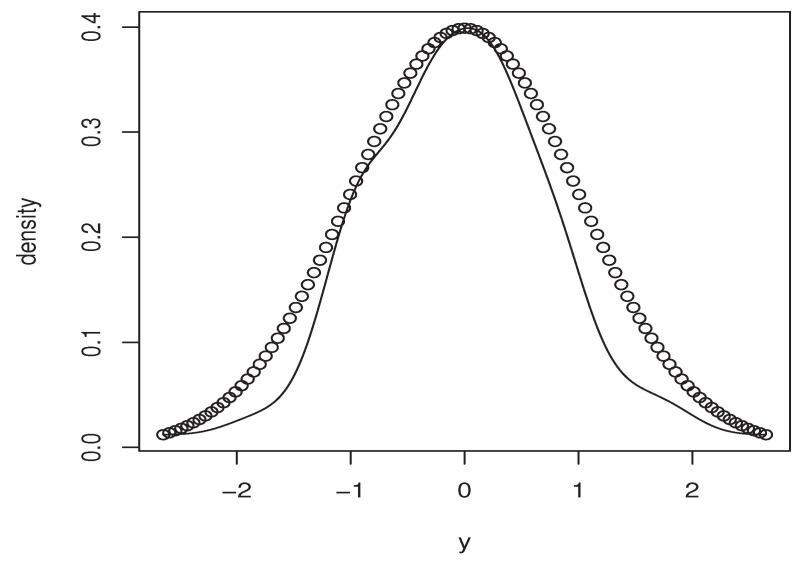
Case (op.norms,γ)=(0.98,0.5).

**Figure 4:**
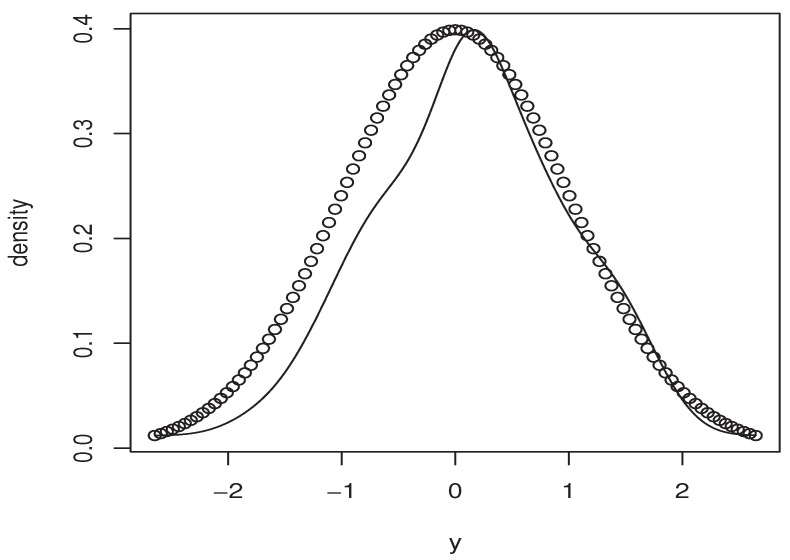
Case (op.norms,γ)=(0.48,5).

**Figure 5:**
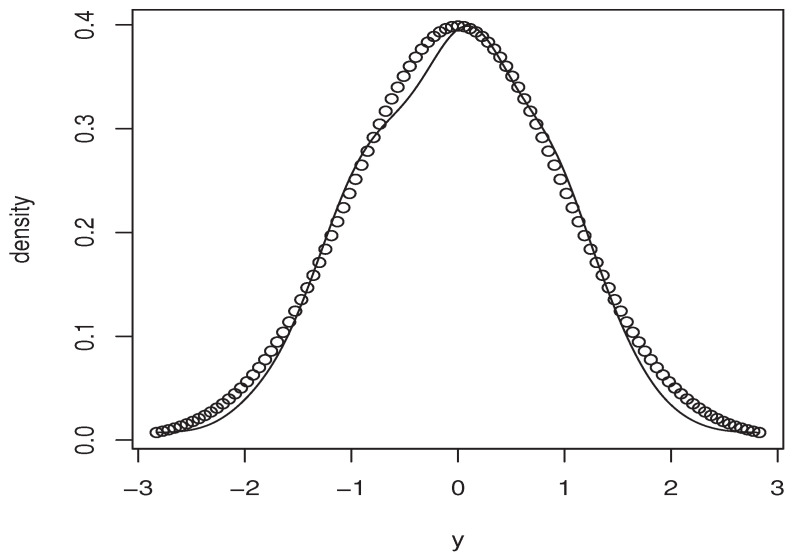
Case (op.norms,γ)=(0.48,0.5).

Now, the *m*-sample of the quantity (9) is calculated using the ideas of the first illustration concerning the construction of $\hat{CDF}\left(y|x\right)$. Moreover, the estimators ${\hat{M}}_{1}$,   ${\hat{M}}_{2}$, and $\hat{P(x)}$ are obtained in the same manner. Specifically, we use the same bandwidth, the same kernel, and the metric associated the FBF with q=0. Of course, the *m*-sample of (9) is drawn for a fixed location curve $x=X_{i_{0}}$ and fixed point $y=Y_{i_{0}}$. The index $i_{0}$ is randomly chosen independently from the sampling process. Furthermore, the behavior of the asymptotic distribution of the quantity (9) is examined by estimating the density of the obtained *m*-sample and we compare it to the density of the standard normal distribution. In order to evaluate the effect of the dependency degree and the missing rate on the accuracy of the asymptotic normality, we perform our sampling process using various values of *op.norms* and γ. Exactly, we keep the same value as that of the first illustration. Finally, we plot in Figure 2, Figure 3, Figure 4 and Figure 5 the estimated density, obtained via the routine code *density* with m=120, against the density of N(0,1). The continuous line represents the estimated density, and the dashed line represents the true density.

Once again, this empirical analysis confirms the theoretical development. In a sense, the estimation approach is strongly impacted by the degree of dependency, as well as the missing rate. Typically, even if the curve of the estimated density is relatively close to the normal density, the accuracy of the asymptotic normality is significantly varied with respect to the values of *op.norms* and γ. It appears that the effect of the missing rate is more important than the degree of dependency. This conclusion is justified by the fact that the missing rate impacts both the bias and the variance terms, whereas the dependency feature impacts only the variance part. To confirm this statement, we report in Table 3 the bias quantities of the different values of (*op.norms*) and γ. The bias term is

$$Bias=\frac{1}{m}\Sigma_{j=1}^{m}{\hat{CDF}}^{j}\left(Y_{i_{0}}|X_{i_{0}}\right)-CDF\left(Y_{i_{0}}|X_{i_{0}}\right),$$

where ${\hat{CDF}}^{j}\left(Y_{i_{0}}|X_{i_{0}}\right)$ is the LLECCDF obtained with the $j^{th}$ sample. The result of Table 3 shows that the effect of the data correlation is too small compared with the variability with respect to the missing rate.

**Figure 6:**
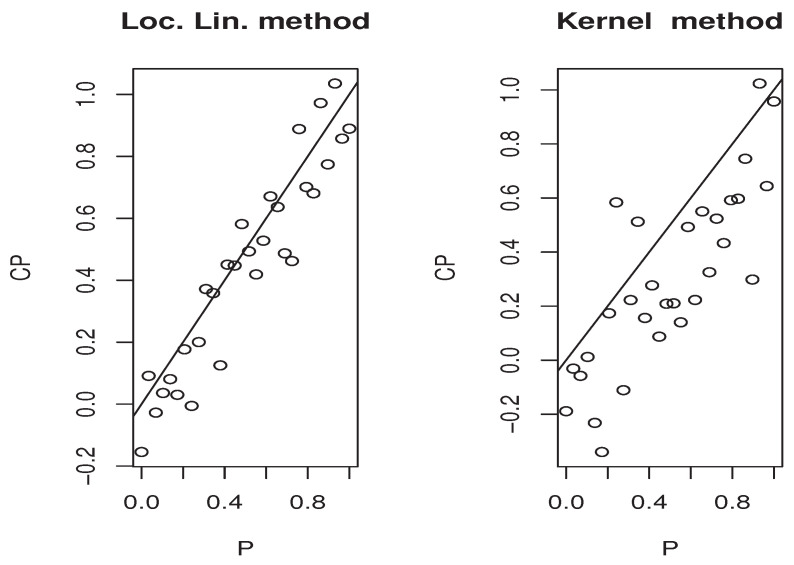
Case (op.norms,γ)=(0.98,5).

**Figure 7:**
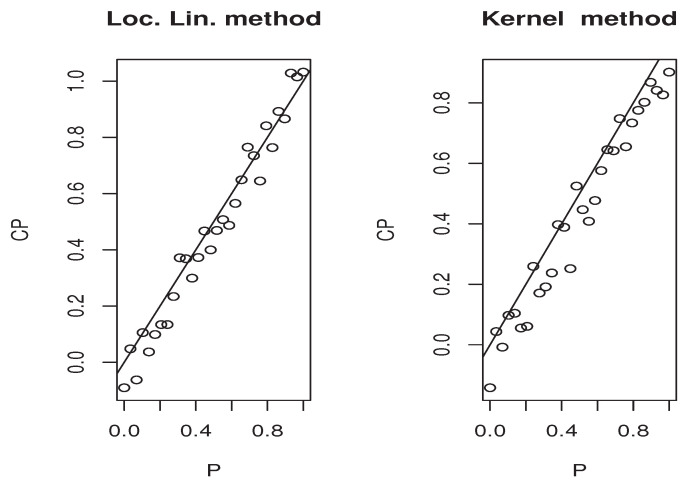
Case (op.norms,γ)=(0.98,0.5).

**Figure 8:**
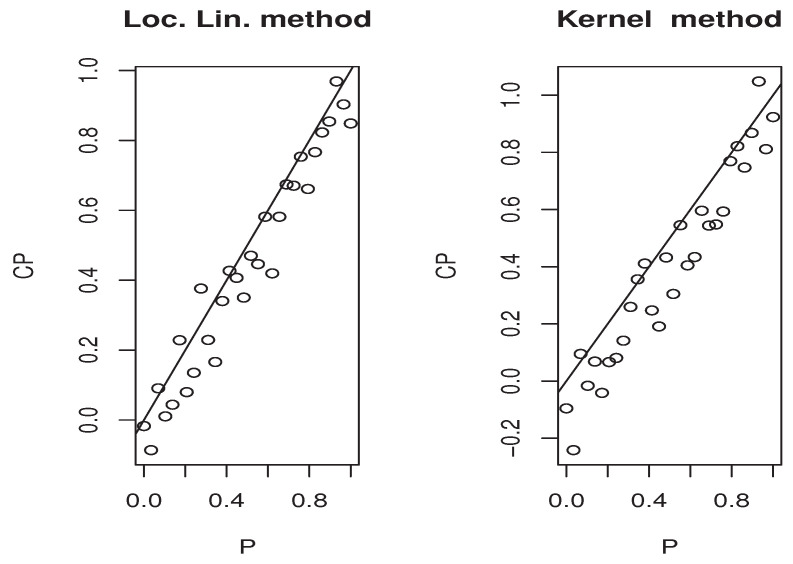
Case (op.norms,γ)=(0.48,5).

**Figure 9:**
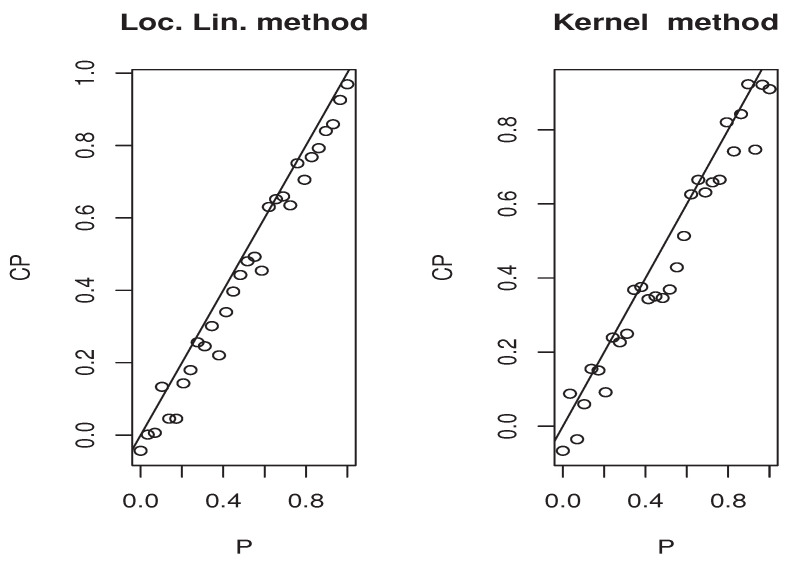
Case (op.norms,γ)=(0.48,0.5).

The third illustration concerns the used estimation method that is the local linear approach. More precisely, we compare it with the classical kernel method. We concentrate on this part in the second model, which is the quantile regression. It is well known that this kind of model has a many scopes of application. One of the important application areas is the prediction problem. At this stage, the quantile regression can be used as single-point predictor when p=0.5 or as a predictive interval $\left[CQF_{p/2},CQF_{1-p/2}\right]$. The latter ensures the existence of the response variable *Y* in $\left[CQF_{p/2}\left(x\right),CQF_{1-p/2}\left(x\right)\right]$ with a probability equal to 1−p. In order to show the easy implantation of the estimator $\hat{CQF_{\alpha }}\left(\cdot \right)$ and to highlight its advantages over the classical kernel method studied by [4], we compare the efficiency of both estimation methods on the construction of the $\left[CQF_{p/2}\left(x\right),CQF_{1-p/2}\left(x\right)\right]$ interval. Undoubtedly, the performance of any predictive interval is measured using two factors that are the coverage probability and the length of the interval. However, for the sake of brevity, we focus in this third illustration only on the coverage probability factor that measures the belonging percentage to the approximated predictive interval. For this aim, we employ the same sampling process as the second illustration, and for each sampling time *j*, we split the observations into learning and testing sample. We determine $\left[{\overset{-}{CQF}}_{p/2}\left(x_{0}\right),{\overset{-}{CQF}}_{1-p/2}\left(x_{0}\right)\right]$ for all point $x_{0}$ in the testing sample, and $\overset{-}{CQF}$ means either the local linear or kernel estimator of the quantile CQF. For the computation aspect, we use the routine code funopare.quantile.lcv for the kernel method, whereas that for the local linear method is obtained by minimizing (3) on a regular grid of 100 points, from $\left(0.9*\min\left(Y_{i}\right),1.1*\max\left(Y_{i}\right)\right)$. To the end, let us point out that we have used the same selection strategies as the previous illustrations, namely, the same bandwidth, the same kernel, and the same FBF metric. In order to give a comprehensive comparison, we examine the efficiency of the predictive interval processing for various values of *p*. Exactly, we examine 30 values of $p_{i}$ in (0,1). Such values are compared with the coverage probabilities of both estimators in the four previous situations covering the strong and weak dependencies, as well as the two levels of missing rates. The results are presented in Figure 6, Figure 7, Figure 8 and Figure 9 for which we plot the different values of $q_{i}=1-p_{i}$ versus the coverage probability $CP_{i}$.

Once again, the estimation quality is strongly affected by the two principal features that are the dependency and the missing phenomena. However, it seems that the local linear algorithm is more robust and more preferment in the sense that its behavior is more stable than the kernel method. To confirm this statement, we summarise in Table 4, the absolute coverage probability error defined by

$$ACPE=\frac{1}{30}\Sigma_{i=1}^{30}\left|CP_{i}-q_{i}\right|,$$

where $CP_{i}$ represents the coverage probability of the predictive interval associated with the threshold $\alpha_{i}$.

## 6. Discussion and Comments

- *Gap between the pointwise and uniform convergence in FDA*: We specify the convergence rate over some known functional structures to highlight the gap between the pointwise and uniform convergence in functional statistics. The first one concerns the resealed version ${\left(W_{c}\left(t\right)=W\left(t/c\right)\right)}_{t\geq 0}$ of Gaussian process $W{\left(t\right)}_{t\geq 0}$. If we assume that the spectral measure μ of *W* such that, for some a>0,
  $$\int e^{a|\lambda |}\mu \left(du\lambda \right)<\infty ,$$
  then the Kolmogorov’s ϵ-entropy of $C_{F}$, the unit ball in reproducing kernel Hilbert spaces of the process $W_{c}\left(\cdot \right)$ as a subset of $\left(C\left(\left[0,1\right]\right),∥\cdot {∥}_{\infty }\right)$, is of the order
  $$\frac{1}{c}\log^{2}\left(\frac{1}{\epsilon }\right);$$
  for instance, see [66], implying an uniform convergence rate over $C_{F}$ asymptotically equal to
  $$O\left(\lambda_{K}^{k_{1}}+\lambda_{J}^{k_{2}}\right)+O_{a.co.}\left({\left(\frac{\ln^{2}n}{n\psi (\lambda_{K})}\right)}^{\frac{1}{2}}\right).$$
  Secondly, if we put $C_{F}$ as the closed unit ball of Cameron–Martin spaces associated with the covariance operator of the standard stationary Ornstein–Uhlenbeck process defined by
  $$Cov\left(s,t\right)=\exp\left(-a|s-t|\right),\;a>0,$$
  the Kolmogorov’s ϵ-entropy of this subset with respect to the norm of the Sobolev space $W^{1,2}\left(\left[0,1\right]\right)$ is of order
  $$\left(\sqrt{(}2a)\left(\frac{1}{\pi \epsilon }\right)\right);$$
  for instance, refer to [66], and the convergence rate is
  $$O\left(\lambda_{K}^{k_{1}}+\lambda_{J}^{k_{2}}\right)+O_{a.co.}\left({\left(\frac{\ln n}{n\psi (\lambda_{K})}\right)}^{\frac{1}{2}}\right).$$
  Thirdly, it is shown in [67] that any closed ball in a Sobolev space $W^{1,1}\left(\left[0,T\right]\right)$ endowed with the norm $L^{1}\left(\left[0,T\right]\right)$ has a Kolmogorov’s ϵ-entropy of the order
  $$\frac{1}{\epsilon }\log\frac{1}{\epsilon },$$
  implying a uniform convergence rate asymptotically equal to
  $$O\left(\lambda_{K}^{k_{1}}+\lambda_{J}^{k_{2}}\right)+O_{a.co.}\left({\left(\frac{\ln^{2}n}{n\psi (\lambda_{K})}\right)}^{\frac{1}{2}}\right).$$
  So, it is clear that the uniform convergence rate differs among functional subsets. However, in the nonfunctional case, where all norms are equivalent, the ϵ-entropy for any compact subset in IR is of the order $\log\left(\frac{1}{\epsilon }\right)$, allowing us to keep the usual convergence rate in the finite-dimensional case, that is
  $$O\left(\lambda_{K}^{k_{1}}+\lambda_{J}^{k_{2}}\right)+O_{a.co.}\left({\left(\frac{\ln n}{n\lambda_{K}}\right)}^{\frac{1}{2}}\right),$$
  which also coincides with the pointwise convergence rate. In conclusion, unlike the finite-dimensional case, there is a real gap between the pointwise and the uniform convergence in FDA. Thus, treating uniform consistency in the FDA is a challenging question; refer to [9,68,69,70].
- *The effect of the basis function on the Kolmogorov’s entropy:*
  Similarly to the previous paragraph, Kolmogorov’s entropy is also affected by the spectral decomposition of the functional variable over specific basis functions. Of course, this relationship is justified by the fact that both tools have similar interpretations. In a sense, the Kolmogorov’s entropy of a given subset $C_{F}$ is the number of binary bits of information required to describe any $x\in C_{F}$ with error ϵ, while the spectral decomposition of $x\in C_{F}$ given a basis functions $\left(f_{i}\right)$ can be viewed as the reconstruction of $x\in C_{F}$. Furthermore, the minimal cardinal of elements of the basis $\left(f_{i}\right)$, sufficient to reconstruct any $x\in C_{F}$ with error ϵ, is so-called the sampling ϵ-entropy of $C_{F}$; for instance, see [71]. Theorem 4.1 in this cited work provides, under some general conditions, that the class of bounded piecewise $C^{k}$ smooth functions as a subspace of $L^{2}$ has a Kolmogorov’s ϵ-entropy of the order
  $${\left(\frac{1}{\epsilon }\right)}^{1/k}.$$
  In contrast, Kolmogorov’s ϵ-entropy of the class of periodic real analytic functions on [−π,π], as a subspace of $C^{0}\left(\left[-\pi ,\pi \right]\right)$ is
  $$\log^{2}\left(\frac{1}{\epsilon }\right).$$
  As a consequence of this statement (see Corollary 4.2 in [71]), the sampling ϵ-entropy of the class spanned by the B-splines basis function is greater than the sampling ϵ-entropy of the class of periodic real analytic functions that is spanned for the Fourier basis function. In conclusion, in practice, the estimator’s accuracy is also affected by the choice of basis functions and the cardinal of the basis used in the metric.
- *Estimation of the unconditional distribution of the MAR response:* One of the fundamental applications of conditional modeling in the MAR structure is the possibility of reconstructing the feature of the MAR variable. In our context, we use the fact that
  $$F_{Y}\left(y\right)=P\left(Y\leq y\right)=I\;E\left[CDF(y|X)\right].$$
  Thus, the natural estimator of the cumulative distribution function is
  $$\hat{F_{Y}}\left(y\right)=\frac{1}{n}\Sigma_{i=1}^{n}\hat{CDF^{-i}}\left(y|X_{i}\right),$$
  where $\hat{CDF^{-i}}\left(y|X_{i}\right)$ is the estimator $\hat{CDF}\left(\cdot |\cdot \right)$ without the observation $\left(X_{i},Y_{i},B_{i}\right)$. An alternative estimator of $\hat{F_{Y}}\left(y\right)$ can be obtained by replacing only the missing observation with the conditional expectation. Specifically, the second estimator is expressed by
  $$\tilde{F_{Y}}\left(y\right)=\frac{1}{n}\Sigma_{i=1}^{n}\left(B_{i}11_{\left[Y_{i}<y\right]}+\left(1-B_{i}\right)\hat{CDF^{-i}}\left(y|X_{i}\right)\right).$$
  We return to [48] for more ideas to construct other estimators. At this stage, the uniform consistency obtained in this paper is an important preliminary tool to derive the root *n*-consistency of this kind of estimator; we refer to [48] for the regression case.

## 7. Concluding Remarks

The convergence rate for a free-distribution functional data analysis is challenging. It requires some advanced tools for functional analysis in pure mathematics. This paper gives several contributions to the existing literature on functional data analysis. First, this paper demonstrated that the Kolmogorov entropy is an essential tool for describing the convergence rate of local linear estimation (LLE). To determine the uniform convergence rate of the LLE of the conditional cumulative distribution function (LLECCDF) and the LLE conditional quantile function (LLECQF), we have used this device. Second, a central limit theorem is established for the proposed estimators. These results are demonstrated under general assumptions that permit coverage of the case of incomplete functional time series. Specifically, we model the dependence using the ergodic assumption and assume that the response variable is missing at random (MAR). Finally, we have evaluated the finite sample performance of the proposed estimators using Monte Carlo simulations. In addition to the previous issues, the present paper opens some important paths for the future. It will be natural to consider in a future investigation of the functional kNN local linear approach quantile regression estimators to obtain an alternative estimator that benefits from the advantages of both methods, the local linear method, and the kNN approach. Extending nonparametric functional concepts to local stationary processes is a relatively underdeveloped field. It would be intriguing to extend our work to the case of the functional local stationary process, which requires nontrivial mathematics; however, doing so would be far beyond the scope of this paper.

## 8. Proofs

This section contains the proof of our results. The notation introduced previously will be employed in the following. All of the proofs rely on applying the exponential inequality of the martingale difference. The proofs are quite lengthy; we limit ourselves to the main arguments.

### 8.1. Proof of the Main Theorems

**Proof of Theorem** **1.** For (7), we write

$$\begin{array}{ccc} {\overset{-}{CDF}}_{N}^{x}\left(y\right) & := & \frac{1}{nI\;E(\Gamma_{1}\left(x\right)Ker_{1}\left(x\right))}\Sigma_{j=1}^{n}I\;E\left(\Gamma_{j}\left(x\right)Ker_{j}\left(x\right)J_{j}\left(x\right)B_{j}|F_{j-1}\right),\\ {\overset{-}{CDF}}_{D}\left(x\right) & := & \frac{1}{nI\;E(\Gamma_{1}\left(x\right)Ker_{1}\left(x\right))}\Sigma_{j=1}^{n}I\;E\left(\Gamma_{j}\left(x\right)Ker_{j}\left(x\right)B_{j}|F_{j-1}\right). \end{array}$$

Then, the statement (7) is based on the following decomposition:

(10) $$\begin{array}{l} \hat{CDF}\left(y|x\right)-CDF\left(y|x\right)\\ \;=B_{n}\left(y|x\right)+\frac{1}{{\hat{CDF}}_{D}\left(x\right)}\left[\left(B_{n}\left(y|x\right)+CDF\left(y|x\right)\right)A_{n}\left(y|x\right)+R_{n}\left(y|x\right)\right], \end{array}$$

where

$$B_{n}\left(y|x\right)=\frac{{\overset{-}{CDF}}_{N}^{x}\left(y\right)}{{\overset{-}{CDF}}_{D}\left(x\right)}-CDF\left(y|x\right),$$

$$A_{n}\left(y|x\right)={\overset{-}{CDF}}_{D}\left(x\right)-{\hat{CDF}}_{D}\left(x\right),$$

and

$$R_{n}\left(y|x\right)={\hat{CDF}}_{N}^{x}\left(y\right)-{\overset{-}{CDF}}_{N}^{x}\left(y\right).$$

So, all that remains is to demonstrate Lemmas 1–3:

**Lemma** **1.** *Under Conditions (H1)–(H5), we have, as n→∞,*

$$\underset{x\in C_{F}}{\sup}\left|B_{n}\left(y|x\right)\right|=O\left(\lambda_{K}^{b_{1}}+\lambda_{J}^{b_{2}}\right).$$

**Lemma** **2.** *Under Conditions (H1)–(H5), we have, as n→∞,*

$$\underset{x\in C_{F}}{\sup}\left|R_{n}\left(y|x\right)\right|=O_{a.co.}\left(\sqrt{\frac{\ln d_{n}}{n\psi (\lambda_{K})}}\right).$$

**Lemma** **3.** *Under Conditions (H1)–(H5), we have, as n→∞,*

$$\underset{x\in C_{F}}{\sup}\left|A_{n}\left(y|x\right)\right|=O_{a.co.}\left(\sqrt{\frac{\ln d_{n}}{n\psi (\lambda_{K})}}\right),$$

*and*

$$\Sigma_{n=1}^{\infty }P\left(\underset{x\in C_{F}}{\inf}{\hat{CDF}}_{D}\left(x\right)<\frac{1}{2}\right)<\infty .$$

□

**Proof of Theorem** **2.** For (8), we define for vector $\vec{\delta }=\left(\begin{array}{c} c\\ d \end{array}\right)$, we put

$$\Phi \left(\vec{\delta }\right)=\alpha -{1\;1}_{Y_{j}\leq \left(c+\beta_{1}\right)+\left(\lambda_{K}^{-1}d+\beta_{2}\right)\alpha_{j}},$$

and we employ Lemma 3 in [30] for

$$V_{n}\left(\vec{\delta },x\right)=\frac{1}{n\psi (\lambda_{K})}\Sigma_{j=1}^{n}\Phi \left(\vec{\delta }\right)\left(\begin{array}{c} 1\\ \lambda_{K}^{-1}\alpha_{j} \end{array}\right)Ker_{j}\left(x\right)B_{j},$$

$$A_{n}\left(x\right)=V_{n}\left(\vec{\delta_{0}},x\right)\;with\;\vec{\delta_{0}}=\left(\begin{array}{c} 0\\ 0 \end{array}\right)\;and\;\delta_{n}=\left(\begin{array}{c} \tilde{\beta_{1}}-\beta_{1}\\ \lambda_{K}\left(\tilde{\beta_{2}}-\beta_{2}\right) \end{array}\right).$$

Thus, (8) is a consequence of Lemmas 4 and 5.

**Lemma** **4.** *Under Conditions (H1)–(H5), we have, as n→∞,*

$$\underset{x\in C_{F}}{\sup}∥A_{n}\left(x\right)∥=O\left(\lambda_{K}^{\min(k_{2},k_{2})}\right)+O_{a.co.}\left({\left(\frac{\ln d_{n}}{n\;\psi (\lambda_{K})}\right)}^{1/2}\right).$$

**Lemma** **5.** *Under Conditions (H1)–(H5), we have, as n→∞,*

$$\begin{array}{l} \underset{x\in C_{F}}{\sup}\underset{∥\vec{\delta }∥\leq M}{\sup}∥V_{n}\left(\vec{\delta },x\right)+\lambda_{0}D\vec{\delta }-A_{n}\left(x\right)∥\\ \;\;=O\left(\lambda_{K}^{\min(k_{2},k_{2})}\right)+O_{a.co.}\left({\left(\frac{\ln d_{n}}{n\;\psi (\lambda_{K})}\right)}^{1/2}\right), \end{array}$$

*where*

$$\lambda_{0}=cdf\left(CQF_{p}\left(x\right)|x\right)P\left(x\right).$$

□

**Proof of Theorem** **3.** Using the decomposition

(11) $$\begin{array}{c} \hat{CDF}\left(y|x\right)-CDF\left(y|x\right)=\frac{C_{n}\left(y|x\right)+Q_{n}\left(y|x\right)}{{\hat{CDF}}_{D}\left(x\right)}+B_{n}\left(y|x\right), \end{array}$$

with

$$Q_{n}\left(y|x\right)=R_{n}\left(y|x\right)+CDF\left(y|x\right)A_{n}\left(y|x\right),$$

and

$$C_{n}\left(y|x\right)=B_{n}\left(y|x\right)A_{n}\left(y|x\right).$$

Then, the asymptotic normality can be demonstrated via the following Lemmas.

**Lemma** **6.** *Under the assumptions of Theorem 3, we have, as n→∞,*

(12) $$\begin{array}{c} \sqrt{\frac{n\psi (\lambda_{K})}{V_{JK}\left(y|x\right)}}Q_{n}\left(y|x\right)\overset{D}{\to }N\left(0,1\right). \end{array}$$

□

### 8.2. Proof of the Technical Lemmas

**Proof of Lemma** **1.** Writing

$$\underset{x\in C_{F}}{\sup}\left|B_{n}\left(y|x\right)\right|=\frac{\underset{x\in C_{F}}{\sup}\left|{\tilde{B}}_{n}\left(y|x\right)\right|}{\underset{x\in C_{F}}{\inf}\left|{\overset{-}{CDF}}_{D}\left(x\right)\right|},$$

where

$${\tilde{B}}_{n}\left(y|x\right)={\overset{-}{CDF}}_{N}^{x}\left(y\right)-CDF\left(y|x\right){\overset{-}{CDF}}_{D}\left(x\right).$$

The latter is

(13) $$\begin{array}{ccc} {\tilde{B}}_{n}\left(y|x\right) & = & \frac{1}{nI\;E(\Gamma_{1}\left(x\right)Ker_{1}\left(x\right))}\Sigma_{j=1}^{n}\left\{I\;E(\Gamma_{j}\left(x\right)Ker_{j}\left(x\right)J_{j}B_{j}|F_{j-1})\right.\\ & & \left.-CDF\left(y|x\right)I\;E(\Gamma_{j}\left(x\right)Ker_{j}\left(x\right)B_{j}|F_{j-1})\right\}\\ & \leq & \frac{1}{nI\;E(\Gamma_{1}\left(x\right)Ker_{1}\left(x\right))}\\ & & \Sigma_{j=1}^{n}\left\{P\left(x\right)I\;E(\Gamma_{j}\left(x\right)Ker_{j}\left(x\right)|I\;E\left[J_{j}\left(y\right)|X_{j}\right]-CDF\left(y|x\right)||F_{j-1})\right\}. \end{array}$$

The integration by part gives

(14) $$I_{Ba(x,\lambda_{K})}\left(X_{j}\right)\left|I\;E\left[J_{j}|X_{j}\right]-CDF\left(y|x\right)\right|\leq C\left(\lambda_{K}^{k_{1}}+\lambda_{J}^{k_{2}}\right).$$

Hence, we obtain

$$\underset{x\in C_{F}}{\sup}\left|{\tilde{B}}_{n}\left(y|x\right)\right|=O\left(\lambda_{K}^{k_{1}}+\lambda_{J}^{k_{2}}\right)\underset{x\in C_{F}}{\sup}{\overset{-}{CDF}}_{D}\left(x\right).$$

This completes the proof. □

**Proof of Lemma** **2.** Firstly, observe that, for all $x\in C_{F}$,

(15) $$|\Gamma_{j}\left(x\right)|\leq nC\lambda_{K}^{2}+nC\lambda_{K}\left|\alpha_{j}\left(x\right)\right|.$$

Next, we let $k\left(x\right)=\arg\min_{k\in \{1,2,\ldots ,d_{n}\}}\left|\delta \right|\left(x,x_{k}\right)$. We have

$$\begin{array}{ccc} \underset{x\in C_{F}}{\sup}\left|R_{n}\left(y|x\right)\right| & \leq & \underset{Q_{1}}{\underset{︸}{\underset{x\in C_{F}}{\sup}|{\hat{CDF}}_{N}^{x_{k}\left(x\right)}\left(y\right)-{\overset{-}{CDF}}_{N}^{x_{k}\left(x\right)}(y)|}}\\ & & +\underset{Q_{2}}{\underset{︸}{\underset{x\in C_{F}}{\sup}|{\hat{CDF}}_{N}^{x}\left(y\right)-{\hat{CDF}}_{N}^{x_{k}\left(x\right)}(y)|}}\\ & & +\underset{Q_{3}}{\underset{︸}{\underset{x\in C_{F}}{\sup}|{\overset{-}{CDF}}_{N}^{x_{k}\left(x\right)}\left(y\right)-{\overset{-}{CDF}}_{N}^{x}(y)|}}. \end{array}$$

We start by treating $Q_{2}$. By using the boundeness on Ker(·) and J(·), we write

$$\begin{array}{ccc} Q_{2} & \leq & \underset{x\in C_{F}}{\sup}\frac{1}{n}\Sigma_{j=1}^{n}|J_{j}\left(y\right)||\frac{1}{I\;E(\Gamma_{1}\left(x\right)Ker_{1}\left(x\right))}\Gamma_{j}\left(x\right)Ker_{j}\left(x\right)B_{j}I_{Ba(x,\lambda_{K})}\left(X_{j}\right)\\ & & -\frac{1}{I\;E(\Gamma_{1}\left(x_{k}\left(x\right)\right)Ker_{1}\left(x_{k}\left(x\right)\right))}\Gamma_{j}\left(x_{k}\left(x\right)\right)B_{j}Ker_{j}\left(x_{k}\left(x\right)\right)I_{Ba(x_{k}\left(x\right),\lambda_{K})}(X_{j})|\\ & \leq & \left(\frac{C}{n^{2}\lambda_{K}^{2}\psi \left(\lambda_{K}\right)}\underset{x\in C_{F}}{\sup}\Sigma_{j=1}^{n}|\Gamma_{j}\left(x\right)B_{j}I_{Ba(x,\lambda_{K})}\left(X_{j}\right)|\times \right.\\ & & \left.|Ker_{j}\left(x\right)-Ker_{j}\left(x_{k}\left(x\right)\right)I_{Ba(x_{k(x)},\lambda_{K})}\left(X_{j}\right)|\right)\\ & & +\left(\frac{C}{n^{2}\lambda_{K}^{2}\psi \left(\lambda_{K}\right)}\underset{x\in C_{F}}{\sup}\Sigma_{j=1}^{n}Ker_{j}\left(x_{k(x)}\right)B_{j}I_{Ba(x_{k(x)},\lambda_{K})}\left(X_{j}\right)\times \right.\\ & & \left.|\Gamma_{j}\left(x\right)I_{Ba(x,\lambda_{K})}\left(X_{j}\right)-\Gamma_{j}\left(x_{k}\left(x\right)\right)|\right)\\ & := & F_{1}+F_{2}. \end{array}$$

By using the fact that the Ker(·) satisfies the Lipschitz condition, we obtain via (15)

$$\begin{array}{l} \left|\Gamma_{j}\left(x\right)|I_{Ba(x,\lambda_{K})}\left(X_{j}\right)|Ker_{j}\left(x\right)-Ker_{j}\left(x_{k}\left(x\right)\right)I_{Ba(x_{k(x)},\lambda_{K})}\left(X_{j}\right)\right|\\ \;\leq \;nC\lambda_{K}^{2}(\frac{r_{n}}{\lambda_{K}}I_{Ba\left(x,\lambda_{K}\right)\cap Ba\left(x_{k(x)},\lambda_{K}\right)}\left(X_{j}\right)\\ \;\;\;\;\;\;+I_{Ba\left(x,\lambda_{K}\right)\cap \bar{Ba(x_{k(x)},\lambda_{K})}}\left(X_{j}\right)), \end{array}$$

from which we infer that

$$\begin{array}{ccc} F_{1} & \leq & \frac{Cr_{n}}{n\lambda_{K}\psi \left(\lambda_{K}\right)}\underset{x\in C_{F}}{\sup}\Sigma_{j=1}^{n}I_{Ba\left(x,\lambda_{K}\right)\cap Ba\left(x_{k(x)},\lambda_{K}\right)}\left(X_{j}\right)B_{j}\\ & & +\frac{C}{n\psi (\lambda_{K})}\underset{x\in C_{F}}{\sup}\Sigma_{j=1}^{n}I_{Ba\left(x,\lambda_{K}\right)\cap \bar{Ba(x_{k(x)},\lambda_{K})}}\left(X_{j}\right)B_{j}. \end{array}$$

Concerning $F_{2}$ we put, for l=0,1 and k=1,2,

$$\begin{array}{ccc} T^{k,l} & = & I_{Ba\left(x_{k(x)},\lambda_{K}\right)\cap Ba\left(x,\lambda_{K}\right)}\left(X_{j}\right)\\ & & \times \left|\left(\Sigma_{i=1}^{n}\alpha_{i}^{k}\left(x\right)Ker_{i}\left(x\right)B_{i}\right)\alpha_{j}^{l}\left(x\right)-\left(\Sigma_{i=1}^{n}\alpha_{i}^{k}\left(x_{k(x)}\right)Ker_{i}\left(x_{k(x)}\right)B_{i}\right)\alpha_{j}^{l}\left(x_{k(x)}\right)\right|. \end{array}$$

Therefore, we obtain

$$T^{k,l}\leq T_{1}^{k,l}+T_{2}^{k,l},$$

where

$$\begin{array}{c} T_{1}^{k,l}=I_{Ba\left(x_{k(x)},\lambda_{K}\right)\cap Ba\left(x,\lambda_{K}\right)}\left(X_{j}\right)\left(\Sigma_{i=1}^{n}|\alpha_{i}^{k}\left(x\right)|Ker_{i}\left(x\right)B_{i}\times |\alpha_{j}^{l}\left(x\right)-\alpha_{j}^{l}\left(x_{k(x)}\right)|\right), \end{array}$$

and

$$\begin{array}{ccc} T_{2}^{k,l} & = & I_{Ba\left(x_{k(x)},\lambda_{K}\right)\cap Ba\left(x,\lambda_{K}\right)}\left(X_{j}\right)(|\alpha_{j}^{l}\left(x_{k(x)}\right)|B_{i}\\ & & \times \left|\Sigma_{i=1}^{n}\left(\alpha_{i}^{k}\left(x\right)Ker_{i}\left(x\right)-\alpha_{i}^{k}\left(x_{k(x)}\right)Ker_{i}\left(x_{k(x)}\right)\right)\right|, \end{array}$$

Making use of the condition (H4), for l=1, we have

$$I_{Ba\left(x_{k(x)},\lambda_{K}\right)\cap Ba\left(x,\lambda_{K}\right)}\left(X_{j}\right)|\alpha_{j}\left(x\right)-\alpha_{j}\left(x_{k(x)}\right)|\leq Cr_{n}I_{Ba\left(x_{k(x)},\lambda_{K}\right)\cap Ba\left(x,\lambda_{K}\right)}\left(X_{j}\right).$$

So, for k=2, l=0,

(16) $$\begin{array}{c} T_{1}^{k,l}=0, \end{array}$$

and for k=1, l=1,

$$\begin{array}{c} T_{1}^{k,l}\leq nCr_{n}\lambda_{K}I_{Ba\left(x_{k(x)},\lambda_{K}\right)\cap Ba\left(x,\lambda_{K}\right)}\left(X_{j}\right). \end{array}$$

Now, for $T_{2}^{k,l}$, we have

$$\begin{array}{ccc} T_{2}^{k,l} & \leq & I_{Ba\left(x_{k(x)},\lambda_{K}\right)\cap Ba\left(x,\lambda_{K}\right)}\left(X_{j}\right)\\ & & \times \left(\Sigma_{i=1}^{n}|\alpha_{j}^{l}\left(x_{k(x)}\right)|Ker_{i}\left(x\right)B_{i}\times |\alpha_{i}^{k}\left(x\right)-\alpha_{i}^{k}\left(x_{k(x)}\right)|\right)\\ & & +I_{Ba\left(x_{k(x)},\lambda_{K}\right)\cap Ba\left(x,\lambda_{K}\right)}\left(X_{j}\right)\\ & & \times \left(\Sigma_{i=1}^{n}|\alpha_{j}^{l}\left(x_{k(x)}\right)||\alpha_{i}^{k}\left(x_{k(x)}\right)|B_{i}\times |Ker_{i}\left(x\right)-Ker_{i}\left(x_{k(x)}\right)|\right). \end{array}$$

We have

$$I_{Ba\left(x_{k(x)},\lambda_{K}\right)\cap Ba\left(x,\lambda_{K}\right)}\left(X_{j}\right)|\alpha_{i}^{2}\left(x\right)-\alpha_{i}^{2}\left(x_{k(x)}\right)|\leq Cr_{n}\lambda_{K}I_{Ba\left(x_{k(x)},\lambda_{K}\right)\cap Ba\left(x,\lambda_{K}\right)}\left(X_{j}\right).$$

This implies that, for k=1,2,

$$I_{Ba\left(x_{k(x)},\lambda_{K}\right)\cap Ba\left(x,\lambda_{K}\right)}\left(X_{j}\right)|\alpha_{i}^{k}\left(x\right)-\alpha_{i}^{k}\left(x_{k(x)}\right)|\leq Cr_{n}\lambda_{K}^{k-1}I_{Ba\left(x_{k(x)},\lambda_{K}\right)\cap Ba\left(x,\lambda_{K}\right)}\left(X_{j}\right).$$

Thus, for (l,k)=(0,2), we have

(17) $$\begin{array}{c} T_{2}^{k,l}\leq nCr_{n}\lambda_{K}I_{Ba\left(x_{k(x)},\lambda_{K}\right)\cap Ba\left(x,\lambda_{K}\right)}\left(X_{j}\right), \end{array}$$

and for l=k=1

(18) $$\begin{array}{c} T_{2}^{k,l}\leq nCr_{n}\lambda_{K}I_{Ba\left(x_{k(x)},\lambda_{K}\right)\cap Ba\left(x,\lambda_{K}\right)}\left(X_{j}\right). \end{array}$$

Combining (16) and (17), we find that

$$\begin{array}{l} I_{Ba\left(x_{k(x)},\lambda_{K}\right)\cap Ba\left(x,\lambda_{K}\right)}\left(X_{j}\right)\left|\left(\Sigma_{i=1}^{n}\alpha_{i}^{2}\left(x\right)Ker_{i}\left(x\right)B_{i}-\alpha_{i}^{2}\left(x_{k(x)}\right)Ker_{i}\left(x_{k(x)}\right)B_{i}\right)\right|\\ \;\;\;\;\;\;\;\;\;\;\leq nCr_{n}\lambda_{K}I_{Ba\left(x_{k(x)},\lambda_{K}\right)\cap Ba\left(x,\lambda_{K}\right)}\left(X_{j}\right), \end{array}$$

and

$$\begin{array}{l} I_{Ba\left(x_{k(x)},\lambda_{K}\right)\cap Ba\left(x,\lambda_{K}\right)}\left(X_{j}\right)\\ \;\times \left|\left(\left(\Sigma_{i=1}^{n}\alpha_{i}\left(x\right)Ker_{i}\left(x\right)B_{i}\right)\alpha_{j}\left(x\right)-\left(\Sigma_{i=1}^{n}\alpha_{i}\left(x_{k(x)}\right)Ker_{i}\left(x_{k(x)}\right)B_{i}\right)\alpha_{j}\left(x_{k(x)}\right)\right)\right|\\ \;\leq \;nCr_{n}\lambda_{K}I_{Ba\left(x_{k(x)},\lambda_{K}\right)\cap Ba\left(x,\lambda_{K}\right)}\left(X_{j}\right). \end{array}$$

Thus, we obtain

$$\begin{array}{ccc} F_{2} & \leq & \frac{Cr_{n}}{n\lambda_{K}\psi \left(\lambda_{K}\right)}\underset{x\in C_{F}}{\sup}\Sigma_{j=1}^{n}I_{Ba\left(x,\lambda_{K}\right)\cap Ba\left(x_{k(x)},\lambda_{K}\right)}\left(X_{j}\right)B_{j}\\ & & +\frac{C}{n\psi (\lambda_{K})}\underset{x\in C_{F}}{\sup}\Sigma_{j=1}^{n}I_{Ba\left(x,\lambda_{K}\right)\cap \bar{Ba(x_{k(x)},\lambda_{K})}}\left(X_{j}\right)B_{j}. \end{array}$$

Consequently, we infer that

$$Q_{2}\leq C\underset{x\in C_{F}}{\sup}\left(Q_{2.1}+Q_{2.2}+Q_{2.3}\right),$$

where

$$\begin{array}{ccc} Q_{2.1} & = & \frac{C}{n\psi (\lambda_{K})}\Sigma_{j=1}^{n}I_{Ba\left(x_{k(x)},\lambda_{K}\right)\cap \bar{Ba(x,\lambda_{K})}}\left(X_{j}\right)B_{j},\\ Q_{2.2} & = & \frac{Cr_{n}}{n\lambda_{K}\psi \left(\lambda_{K}\right)}\Sigma_{j=1}^{n}I_{Ba\left(x,\lambda_{K}\right)\cap Ba\left(x_{k(x)},\lambda_{K}\right)}\left(X_{j}\right)B_{j},\\ Q_{2.3} & = & \frac{C}{n\psi (\lambda_{K})}\Sigma_{j=1}^{n}I_{Ba\left(x,\lambda_{K}\right)\cap \bar{Ba(x_{k(x)},\lambda_{K})}}\left(X_{j}\right)B_{j}. \end{array}$$

Now, we apply an exponential inequality for the difference martingale random variables of $Z_{j}$ that are defined by

$$\begin{array}{c} Z_{j}=\left\{\begin{array}{cc} \frac{1}{\psi (\lambda_{K})}\left[I_{Ba\left(x_{k(x)},\lambda_{K}\right)\cap \bar{Ba(x,\lambda_{K})}}\left(X_{j}\right)\right]B_{j} & \mathrm{for}\;Q_{1.1},\\ \frac{r_{n}}{\lambda_{K}\psi \left(\lambda_{K}\right)}\left[I_{Ba\left(x,\lambda_{K}\right)\cap \bar{Ba(x_{k(x)},\lambda_{K})}}\left(X_{j}\right)\right]B_{j} & \mathrm{for}\;Q_{1.2},\\ \frac{1}{\psi_{K}}\left[I_{Ba\left(x,\lambda_{K}\right)\cap \bar{Ba(x_{k(x)},\lambda_{K})}}\left(X_{j}\right)\right]B_{j} & \mathrm{for}\;Q_{1.3}. \end{array}\right. \end{array}$$

Keep in mind that

$$\begin{array}{ccc} Z_{j} & = & O\left(\frac{1}{\psi (\lambda_{K})}\right),\\ I\;E[Z_{j}|F_{j-1}] & = & O\left(\frac{r_{n}}{\psi (\lambda_{K})}\right)\\ I\;E[Z_{j}^{2}||F_{j-1}] & = & O\left(\frac{r_{n}}{\psi {\left(\lambda_{K}\right)}^{2}}\right). \end{array}$$

Therefore, we infer

$$Q_{2.1}=O\left(\frac{r_{n}}{\psi (\lambda_{K})}\right)+O_{a.co.}\left(\sqrt{\frac{r_{n}\ln n}{n\psi {\left(\lambda_{K}\right)}^{2}}}\right),$$

and

$$Q_{2.2}=O_{a.co.}\left(\sqrt{\frac{\ln d_{n}}{n\psi (\lambda_{K})}}\right).$$

We deduce

$$Q_{2}=O_{a.co.}\left(\sqrt{\frac{\ln d_{n}}{n\psi (\lambda_{K})}}\right),$$

and ∀ε>0, we obtain

$$\begin{array}{l} I\;P\left(Q_{1}>\varepsilon \sqrt{\frac{lnd_{n}}{n\psi (\lambda_{K})}}\right)\\ \;\;=\;I\;P\left(\max_{k\in 1,\ldots ,d_{n}}|{\hat{CDF}}_{N}^{x_{k}\left(x\right)}\left(y\right)-{\overset{-}{CDF}}_{N}^{x_{k}\left(x\right)}\left(y\right)|>\varepsilon \right)\\ \;\;\;\;\;\;\;\leq \;d_{n}\max_{k\in 1,\ldots ,d_{n}}I\;P\left({\hat{CDF}}_{N}^{x_{k}\left(x\right)}\left(y\right)-{\overset{-}{CDF}}_{N}^{x_{k}\left(x\right)}\left(y\right)|>\varepsilon \sqrt{\frac{\ln d_{n}}{n\psi (\lambda_{K})}}\right). \end{array}$$

Let

$${\hat{CDF}}_{N}^{x_{k}\left(x\right)}\left(y\right)-{\overset{-}{CDF}}_{N}^{x_{k}\left(x\right)}\left(y\right)=\frac{1}{nI\;E(\Gamma_{1}\left(x\right)Ker_{1}\left(x\right))}\Sigma_{j=1}^{n}S_{j},$$

where

$$S_{j}=\Gamma_{j}\left(x_{k(x)}\right)Ker_{j}\left(x_{k(x)}\right)J_{j}\left(y\right)B_{j}-I\;E\left(\Gamma_{j}\left(x_{k(x)}\right)Ker_{j}\left(x_{k(x)}\right)J_{j}\left(y\right)B_{j}|F_{j-1}\right).$$

We readily obtain

$$I\;E\left(S_{j}^{2}|F_{j-1}\right)\leq 2C^{{}^{\prime }}n^{2}\lambda_{K}^{4}\psi_{j}\left(\lambda_{K}\right).$$

Now, for all ε>0, we infer that

$$\begin{array}{l} I\;P\left(|{\hat{CDF}}_{N}^{x_{k}\left(x\right)}\left(y\right)-{\overset{-}{CDF}}_{N}^{x_{k}\left(x\right)}\left(y\right)|>\varepsilon \sqrt{\frac{\ln d_{n}}{n\psi (\lambda_{K})}}\right)\\ \;\;\leq I\;P\left(|\frac{1}{nI\;E(\Gamma_{1}Ker_{1})}\Sigma_{j=1}^{n}S_{j}|>\varepsilon \sqrt{\frac{\ln d_{n}}{n\psi (\lambda_{K})}}\right)\\ \;\;\leq 2\exp^{-C_{0}\varepsilon^{2}\ln d_{n}}. \end{array}$$

Choosing ε for which $C_{0}\varepsilon^{2}=\varsigma$, we then infer

$$d_{n}\max_{k\in 1,\ldots ,d_{n}}I\;P\left(|{\hat{CDF}}_{N}^{x_{k}\left(x\right)}\left(y\right)-{\overset{-}{CDF}}_{N}^{x_{k}\left(x\right)}\left(y\right)|>\varepsilon \sqrt{\frac{\ln d_{n}}{n\psi (\lambda_{K})}}\right)\leq C^{{}^{\prime }}d_{n}^{1-\varsigma }.$$

Since $\Sigma_{n=1}^{\infty }d_{n}^{1-\varsigma }<\infty ,$ we obtain that

$$Q_{1}=O_{a.co.}\left(\sqrt{\frac{\ln d_{n}}{n\psi (\lambda_{K})}}\right).$$

For $Q_{3}$, we have

$$Q_{3}\leq I\;E\left(\underset{x\in C_{F}}{\sup}\left|{\hat{CDF}}_{N}^{x_{k}\left(x\right)}\left(y\right)-{\overset{-}{CDF}}_{N}^{x_{k}\left(x\right)}\left(y\right)\right||F_{j-1}\right).$$

We follow the proof for $Q_{1}$ to obtain

$$Q_{3}=O_{a.co.}\left(\sqrt{\frac{\ln d_{n}}{n\psi (\lambda_{K})}}\right),$$

which achieves the demonstration of Lemma 2. □

**Proof of Lemma** **3.** Obviously, the first statement is deduced by taking $J_{j}=1$ in Lemma 2, while for the second result, we use

$$\underset{x\in C_{F}}{\inf}|{\hat{CDF}}_{D}\left(x\right)|\leq \frac{\underset{x\in C_{F}}{\inf}P\left(x\right)}{2},$$

meaning that there exists $x\in C_{F}$ such that

$$\begin{array}{c} P\left(x\right)-{\hat{CDF}}_{D}\left(x\right)\geq \frac{\underset{x\in C_{F}}{\inf}P\left(x\right)}{2}\\ \;\;\;⟹\underset{x\in C_{F}}{\sup}|P\left(x\right)-{\hat{CDF}}_{D}\left(x\right)|\geq \frac{\underset{x\in C_{F}}{\inf}P\left(x\right)}{2}. \end{array}$$

So, we have

$$\begin{array}{l} I\;P\left(\underset{x\in C_{F}}{\inf}|{\hat{CDF}}_{D}\left(x\right)\leq \frac{1}{2}\right)\\ \;\;\;\leq \;\;\;\;I\;P\left(\underset{x\in C_{F}}{\sup}|P\left(x\right)-{\hat{CDF}}_{D}\left(x\right)|\geq \frac{\underset{x\in C_{F}}{\inf}P\left(x\right)}{2}\right).\\ \;\;\;\leq \;\;\;\;I\;P\left(\underset{x\in C_{F}}{\sup}|I\;E({\hat{CDF}}_{D}\left(x\right)-{\hat{CDF}}_{D}\left(x\right)|\geq \frac{\underset{x\in C_{F}}{\inf}P\left(x\right)}{2}\right). \end{array}$$

Consequently,

$$\Sigma_{i=1}^{\infty }I\;P\left(\underset{x\in C_{F}}{\inf}|{\hat{CDF}}_{D}\left(x\right)|\leq \frac{\underset{x\in C_{F}}{\inf}P\left(x\right)}{2}\right)<\infty .$$

Hence, the proof is complete. □

**Proof of Lemma** **4.** Notice that

$$\underset{x\in C_{F}}{\sup}∥A_{n}\left(x\right)∥\leq \underset{x\in C_{F}}{\sup}∥A_{n}\left(x\right)-{\overset{-}{A}}_{n}\left(x\right)∥+\underset{x\in C_{F}}{\sup}∥{\overset{-}{A}}_{n}\left(x\right)∥,$$

where

$${\overset{-}{A}}_{n}\left(x\right):=\frac{1}{n\psi (\lambda_{K})}\Sigma_{j=1}^{n}I\;E\left(\Phi \left(\vec{\delta_{0}}\right)\left(\begin{array}{c} 1\\ \lambda_{K}^{-1}\alpha_{j} \end{array}\right)Ker_{j}\left(x\right)B_{j}|F_{j-1}\right).$$

So, all that remains is to prove that

(19) $$\underset{x\in C_{F}}{\sup}∥A_{n}\left(x\right)-{\overset{-}{A}}_{n}\left(x\right)∥=O_{a.co.}\left({\left(\frac{\ln d_{n}}{n\;\psi (\lambda_{K})}\right)}^{1/2}\right)$$

and

(20) $$\underset{x\in C_{F}}{\sup}∥{\overset{-}{A}}_{n}\left(x\right)∥=O\left(\lambda_{K}^{\min(k_{2},k_{2})}\right).$$

The proof of (19) follows the same lines as the Lemma 2, while (20) is based on the same idea as in Lemma 2 using the fact that

$$I_{Ba(x,\lambda_{K})}\left(X_{1}\right)\left|CDF\left(CQF_{p}\left(x\right)|x\right)-CDF\left(\beta_{1}+\beta_{2}\alpha_{1}|X_{1}\right)\right|\leq C\left(\lambda_{K}^{\min(k_{1},k_{2})}\right).$$

This completes the proof of Lemma 4. □

**Proof of Lemma** **5.** We prove that

(21) $$\underset{x\in C_{F}}{\sup}\underset{∥\delta ∥\leq M}{\sup}∥V_{n}\left(\vec{\delta },x\right)-A_{n}\left(x\right)-\left({\overset{-}{V}}_{n}\left(\vec{\delta },x\right)-{\overset{-}{A}}_{n}\left(x\right)\right)∥=O_{a.co.}\left(\sqrt{\frac{\log n}{n\psi (\lambda_{K})}}\right),$$

and

(22) $$\underset{x\in C_{F}}{\sup}\underset{∥\delta ∥\leq M}{\sup}∥\left({\overset{-}{V}}_{n}\left(\vec{\delta },x\right)-{\overset{-}{A}}_{n}\left(x\right)\right)+cdf\left(CQF_{p}\left(x\right)|x\right)D\delta ∥=O\left(\lambda_{K}^{\min(k_{1},k_{2})}\right),$$

where

$${\overset{-}{V}}_{n}\left(\vec{\delta },x\right):=\frac{1}{n\psi (\lambda_{K})}\Sigma_{j=1}^{n}I\;E\left(\Phi \left(\vec{\delta }\right)(\begin{array}{c} 1\\ \lambda_{K}^{-1}\alpha_{j} \end{array}\right)Ker_{j}\left(x\right)B_{j}|F_{j-1}).$$

In order to show (21), we keep the definition of k(x) as in Lemma 2, we use the compactness of the ball B(0,M) in ${I\;R}^{2}$, and we write

$$B\left(0,M\right)\subset \overset{d_{n}}{\underset{j=1}{⋃}}B\left(\delta_{j},l_{n}\right),\;\delta_{j}=\left(\begin{array}{c} c_{j}\\ d_{j} \end{array}\right)\;\mathrm{and}\;l_{n}=d_{n}^{-1}=1/\sqrt{n}.$$

Then, we take $j\left(\delta \right)=\arg\min_{j}\left|\delta -\delta_{j}\right|$. Similarly to Lemma 2 we write

$$\begin{array}{l} \;\underset{x\in C_{F}}{\sup}\underset{∥\delta ∥\leq M}{\sup}∥V_{n}\left(\vec{\delta },x\right)-A_{n}\left(x\right)-\left({\overset{-}{V}}_{n}\left(\vec{\delta },x\right)-{\overset{-}{A}}_{n}\left(x\right)\right)∥\\ \;\leq \;\;\underset{T_{1}}{\underset{︸}{\underset{x\in C_{F}}{\sup}\underset{∥\delta ∥\leq M}{\sup}∥V_{n}\left(\vec{\delta },x\right)-A_{n}\left(x\right)-\left(V_{n}\left(\vec{\delta },x_{k(x)}\right)-A_{n}\left(x_{k(x)}\right)\right)∥}}\\ \;+\underset{T_{2}}{\underset{︸}{\underset{x\in C_{F}}{\sup}\underset{∥\delta ∥\leq M}{\sup}∥V_{n}\left(\vec{\delta },x_{k(x)}\right)-V_{n}\left({\vec{\delta }}_{j(\delta )},x_{k(x)}\right)∥}}\\ \;+\underset{T_{3}}{\underset{︸}{\underset{x\in C_{F}}{\sup}\underset{∥\delta ∥\leq M}{\sup}∥V_{n}\left({\vec{\delta }}_{j(\delta )},x_{k(x)}\right)-A_{n}\left(x_{k(x)}\right)-\left({\overset{-}{V}}_{n}\left({\vec{\delta }}_{j(\delta )},x_{k(x)}\right)-{\overset{-}{A}}_{n}\left(x_{k(x)}\right)\right)∥}}\\ \;+\underset{T_{4}}{\underset{︸}{\underset{x\in C_{F}}{\sup}\underset{∥\delta ∥\leq M}{\sup}∥{\overset{-}{V}}_{n}\left({\vec{\delta }}_{j(\delta )},x_{k(x)}\right)-{\overset{-}{V}}_{n}\left(\vec{\delta },x_{k(x)}\right)∥}}\\ \;+\underset{T_{5}}{\underset{︸}{\underset{x\in C_{F}}{\sup}\underset{∥\delta ∥\leq M}{\sup}∥{\overset{-}{V}}_{n}\left(\vec{\delta },x_{k(x)}\right)-{\overset{-}{A}}_{n}\left(x_{k(x)}\right)-\left({\overset{-}{V}}_{n}\left(\vec{\delta },x\right)-{\overset{-}{A}}_{n}\left(x\right)\right)∥}}. \end{array}$$

We treat $T_{1}$ and $T_{2}$ as $Q_{2}$ in Lemma 2. Meanwhile, $T_{4}$ and $T_{5}$ are evaluated as in $Q_{3}$. Finally, we use the idea of $Q_{1}$ to evaluate $T_{3}$. The statement (22) is a consequence of

$$\lambda_{K}^{-a}I\;E\left[\alpha_{i}^{-a}Ker_{i}^{b}\right]=\psi \left(\lambda_{K}\right)\left(Ker^{b}\left(1\right)-\int_{-1}^{1}{\left(u^{a}Ker^{b}\left(u\right)\right)}^{\prime }\Psi \left(u\right)du\right)+o\left(\psi \left(\lambda_{K}\right)\right).$$

Hence, the proof is complete. □

**Proof of Lemma** **1.** For a fixed *x*, we put all j=1,…,n,$\left(\Gamma_{1}\left(x\right),Ker_{1}\left(x\right)\right)=\left(\Gamma_{1},Ker_{1}\right)$. Next, let us denote

$$\eta_{n,j}=\frac{\sqrt{n\psi (\lambda_{K})}}{nI\;E(\Gamma_{1}Ker_{1})}\left(J_{j}-CDF\left(y|x\right)\right)\Gamma_{j}Ker_{j}B_{j},$$

and we define $\xi_{n,j}=\eta_{n,j}-I\;E\left(\eta_{n,j}|F_{j-1}\right).$ It is clear that

(23) $$\begin{array}{c} \sqrt{n\psi (\lambda_{K})}Q_{n}\left(y|x\right)=\Sigma_{j=1}^{n}\xi_{n,j}. \end{array}$$

Therefore, to demonstrate Lemma 6, we have to prove the following:

(24) $$\Sigma_{j=1}^{n}I\;E\left(\xi_{n,j}^{2}|F_{j-1}\right)\overset{P}{\to }V_{JK}\left(y|x\right),$$

and

(25) $$\forall \varepsilon >0\;\;nI\;E\left(\xi_{n,j}^{2}I_{\left[|\xi_{n,j}^{2}|>\varepsilon \right]}\right)=o\left(1\right).$$

For (24), we have

$$I\;E\left(\xi_{n,j}^{2}|F_{j-1}\right)=I\;E\left(\eta_{n,j}^{2}|F_{j-1}\right)-{\left(I\;E\left(\eta_{n,j}|F_{j-1}\right)\right)}^{2}.$$

Thus, it suffices to show that

(26) $$\lim_{n\to \infty }\Sigma_{j=1}^{n}{\left(I\;E\left(\eta_{n,j}|F_{j-1}\right)\right)}^{2}=0\;\mathrm{in}\;\mathrm{probability},$$

and

(27) $$\lim_{n\to \infty }\Sigma_{j=1}^{n}I\;E\left(\eta_{n,j}^{2}|F_{j-1}\right)=V_{JK}\left(y|x\right)\;\mathrm{in}\;\mathrm{probability}.$$

For (26), we use Lemma 5 of [72] to conclude

$$\begin{array}{ccc} \left|I\;E(\eta_{n,j}|F_{j-1})\right| & = & \frac{\sqrt{n\psi (\lambda_{K})}}{nI\;E(\Gamma_{1}Ker_{1}}\left|I\;E\left(B_{j}\left(J_{j}-CDF\left(y|x\right)\right)\Gamma_{j}Ker_{j}|F_{j-1}\right)\right|\\ & \leq & C\sqrt{n\psi (\lambda_{K})}\left(\lambda_{K}^{b_{1}}+\lambda_{J}^{b_{2}}\right)\frac{1}{n\psi (\lambda_{K})}\left(P\left(x\right)+o\left(1\right)\right). \end{array}$$

Thus, we have

$$\Sigma_{j=1}^{n}{\left(I\;E\left(\eta_{n,j}|F_{j-1}\right)\right)}^{2}=O_{a.co.}\left(n\psi \left(\lambda_{K}\right){\left(\lambda_{K}^{b_{1}}+\lambda_{J}^{b_{2}}\right)}^{2}\right).$$

For Equation (27), we have

$$\begin{array}{l} \Sigma_{j=1}^{n}I\;E\left(\eta_{n,j}^{2}|F_{j-1}\right)\\ \;\;=\;\;\frac{\psi (\lambda_{K})}{n{\left(I\;E\left(\Gamma_{1}Ker_{1}\right)\right)}^{2}}\Sigma_{j=1}^{n}I\;E\left(\Gamma_{j}^{2}Ker_{j}^{2}B_{j}{\left(J_{j}-CDF\left(y|x\right)\right)}^{2}|F_{j-1}\right)\\ \;\;=\;\;\frac{\psi (\lambda_{K})}{n{\left(I\;E\left(\Gamma_{1}Ker_{1}\right)\right)}^{2}}\Sigma_{j=1}^{n}I\;E\left(\Gamma_{j}^{2}Ker_{j}^{2}B_{j}I\;E\left[{\left(J_{j}-CDF\left(y|x\right)\right)}^{2}|X_{j}\right]|F_{j-1}\right). \end{array}$$

Since

$$\begin{array}{ccc} I\;E[{\left(J_{j}-CDF\left(y|x\right)\right)}^{2}|X_{j}] & = & \mathrm{Var}\left[J_{j}|X_{j}\right]+{\left[I\;E\left(J_{j}|X_{j}\right)-CDF\left(y|x\right)\right]}^{2}, \end{array}$$

we have

(28) $$\begin{array}{c} \mathrm{Var}\left[J_{j}|X_{j}\right]=I\;E\left(J_{j}^{2}|X_{j}\right)-{\left(I\;E\left(J_{j}|X_{j}\right)\right)}^{2}. \end{array}$$

We highlight that

(29) $$\begin{array}{ccc} I\;E[J_{j}|X_{j}] & = & \int_{I\;R}J^{\left(1\right)}\left(t\right)\left[CDF\left(y-\lambda_{J}t|x\right)-CDF\left(y|x\right)\right]dt+CDF\left(y|x\right)\\ & = & CDF(y|x), \end{array}$$

and

$$\begin{array}{ccc} I\;E[J_{j}^{2}|X_{j}] & = & \int_{I\;R}J^{2}\left(\frac{y-z}{\lambda_{J}}\right)cdf\left(z|x\right)dz\\ & = & \int_{I\;R}2J\left(t\right)J^{\left(1\right)}\left(t\right)\left[CDF\left(y-\lambda_{J}t|x\right)-CDF\left(y|x\right)\right]dt\\ & & +\int_{I\;R}2J^{\left(1\right)}\left(t\right)J\left(t\right)CDF\left(y|x\right)dt. \end{array}$$

Using the fact

$$\int_{I\;R}J^{\left(1\right)}\left(t\right)J\left(t\right)CDF\left(y|x\right)dt=CDF\left(y|x\right),$$

we readily obtain, as n→∞,

(30) $$\begin{array}{c} I\;E\left[J_{j}^{2}|X_{j}\right]⟶CDF\left(y|x\right). \end{array}$$

Combining (29) with (30), we arrive at

(31) $$\begin{array}{c} \mathrm{Var}\left[J_{j}|X_{j}\right]=CDF\left(y|x\right)\left(1-CDF\left(y|x\right)\right). \end{array}$$

Concerning $\beta_{n_{2}},$ we obtain from (29) that, as n→∞,

$$\begin{array}{c} \beta_{n_{2}}⟶0. \end{array}$$

Thereafter, we obtain

$$\begin{array}{ccc} \Sigma_{j=1}^{n}I\;E\left(\eta_{n,j}^{2}|F_{j-1}\right) & = & \frac{\psi (\lambda_{K})}{n{\left(I\;E\left(\Gamma_{1}Ker_{1}\right)\right)}^{2}}\Sigma_{j=1}^{n}I\;E\left(\Gamma_{j}^{2}K_{j}^{2}B_{j}\beta_{n_{1}}|F_{j-1}\right)\\ & = & \frac{\psi (\lambda_{K})}{n{\left(I\;E\left(\Gamma_{1}Ker_{1}\right)\right)}^{2}}\Sigma_{j=1}^{n}I\;E\left(\Gamma_{j}^{2}K_{j}^{2}I\;E\left(B_{j}|X_{j}\right)\beta_{n_{1}}|F_{j-1}\right)\\ & = & \frac{\psi (\lambda_{K})}{n{\left(I\;E\left(\Gamma_{1}Ker_{1}\right)\right)}^{2}}\Sigma_{j=1}^{n}I\;E\left(\left(P\left(x\right)+o\left(1\right)\right)\Gamma_{j}^{2}K_{j}^{2}\beta_{n_{1}}|F_{j-1}\right). \end{array}$$

The use of Equation (31) allow us to obtain, as n→∞,

$$\begin{array}{ccc} & & \Sigma_{j=1}^{n}I\;E\left(\eta_{n,j}^{2}|F_{j-1}\right)⟶\frac{M_{2}}{M_{1}^{2}}P\left(x\right)\left(1-CDF\left(y|x\right)\right)CDF\left(y|x\right)\\ & & =V_{JK}\left(y|x\right); \end{array}$$

this last part completes the proof of our first claim. Concerning (25), we have

$$nI\;E\left(\xi_{n,j}^{2}\right)\leq 4nI\;E\left(\eta_{n,j}^{2}I_{\left[|\eta_{n,j}|>\frac{\varepsilon }{2}\right]}\right).$$

By Markov–Hölder’s inequalities, we obtain ε>0:

$$I\;E\left(\eta_{n,j}^{2}I_{\left[|\eta_{n,j}|>\frac{\varepsilon }{2}\right]}\right)\leq \frac{I\;E(|\eta_{n,j}{\left|\right)}^{2a}}{{\left(\varepsilon /2\right)}^{2a/b}}.$$

Putting $a=1+\frac{\delta^{\prime }}{2}$ for $\delta^{\prime }>0$ and by recalling that the function

$$G_{2+\delta^{\prime }}=I\;E(|J_{j}{-CDF\left(y|x\right)|}^{2+\delta^{\prime }}\left|X_{j}\right)$$

is continuous, we have

$$\begin{array}{ccc} 4nI\;E(\eta_{n,j}^{2}I_{\left[|\eta_{n,j}|>\frac{\varepsilon }{2}\right]}) & \leq & C{\left(\frac{\psi (\lambda_{K})}{n}\right)}^{\frac{2+\delta^{\prime }}{2}}\frac{n}{{\left(I\;E\left(\Gamma_{1}Ker_{1}\right)\right)}^{2+\delta^{\prime }}}\\ & & \times I\;E([|\left(J_{j}-CDF\left(y|x\right)\right)|\Gamma_{j}Ker_{j}B_{j}{]}^{2+\delta^{\prime }})\\ & \leq & C{\left(\frac{\psi (\lambda_{K})}{n}\right)}^{\frac{2+\delta^{\prime }}{2}}\frac{n}{{\left(I\;E\left(\Gamma_{1}Ker_{1}\right)\right)}^{2+\delta^{\prime }}}\\ & & \times I\;E(P\left(x\right)|\Gamma_{j}Ker_{j}{|}^{2+\delta^{\prime }}[I\;E\left(|J_{j}-CDF\left(y|x\right)\right){|}^{2+\delta^{\prime }}|X_{j}])\\ & \leq & C\left(P\left(x\right)+o\left(1\right)\right){\left(\frac{\psi (\lambda_{K})}{n}\right)}^{\frac{2+\delta^{\prime }}{2}}\frac{n}{{\left(I\;E\left(\Gamma_{1}Ker_{1}\right)\right)}^{2+\delta^{\prime }}}\\ & & \times I\;E(|\Gamma_{j}Ker_{j}{|}^{2+\delta^{\prime }})G_{2+\delta^{\prime }}\\ & = & O\left({\left(n\psi \left(\lambda_{K}\right)\right)}^{\frac{-\delta^{\prime }}{2}}\right)⟶0,\;\;\;\mathrm{as}\;n\to \infty . \end{array}$$

Hence, the proof is complete. □

## Figures and Tables

**Table 1:** MSE error for LLECCDF.

The Metric Type	*op.norms*	γ	*q* or *m*	MSE
Fourier Basis Functions				
	0.98	5	2	0.196
			0	0.193
		0.5	2	0.184
			0	0.187
		0.05	2	0.156
			0	0.155
	0.48	5	2	0.172
			0	0.174
		0.5	2	0.168
			0	0.161
		0.05	2	0.149
			0	0.151
B-spline Basis Functions				
	0.48	5	2	0.266
			0	0.264
		0.5	2	0.257
			0	0.259
		0.05	2	0.251
			0	0.248
	0.08	5	2	0.243
			0	0.242
		0.5	2	0.239
			0	0.237
		0.05	2	0.227
			0	0.230
PCA-metric				
	0.98	5	3	0.408
			1	0.407
		0.5	3	0.398
			1	0.394
		0.05	3	0.387
			1	0.376
	0.08	5	3	0.143
			1	0.292
		0.5	3	0.295
			1	0.283
		0.05	3	0.284
			1	0.279

**Table 2:** MSE error for LLECQF.

The Metric Type	*op.norms*	γ	*q* or *m*	MSE
Fourier Basis Functions				
	0.98	5	2	0.091
			0	0.093
		0.5	2	0.081
			0	0.085
		0.05	2	0.056
			0	0.054
	0.08	5	2	0.051
			0	0.04
		0.5	2	0.033
			0	0.039
		0.05	2	0.031
			0	0.023
B-spline Basis Functions				
	0.98	5	2	0.128
			0	0.123
		0.5	2	0.121
			0	0.122
		0.05	2	0.117
			0	0.114
	0.48	5	2	0.109
			0	0.107
		0.5	2	0.099
			0	0.095
		0.05	2	0.085
			0	0.074
PCA-metric				
	0.48	5	3	0.104
			1	0.108
		0.5	3	0.102
			1	0.101
		0.05	3	0.099
			1	0.097
	0.08	5	3	0.086
			1	0.092
		0.5	3	0.081
			1	0.078
		0.05	3	0.064
			1	0.061

**Table 3:** Bias error for LLECQF.

*op.norms*	γ	Bias
0.98	5	0.121
	0.5	0.093
0.48	5	0.109
	0.5	0.091

**Table 4:** Coverage probability error.

Method	*op.norms*	γ	ACPE
Local linear estimator	0.98	5	0.051
		0.5	0.042
	0.48	5	0.044
		0.5	0.037
Kernel method	0.98	5	0.19
		0.5	0.098
	0.48	5	0.13
		0.5	0.067

## Data Availability

Not applicable.
